# Characterising oral microbial signatures for periodontal disease in the NHANES population

**DOI:** 10.2340/aos.v85.45662

**Published:** 2026-03-30

**Authors:** Zhaocheng Zhuang, Yangjia Chen, Yongjin Wang, Donghong Wei

**Affiliations:** a Department of Preventive Medicine, School of Health, Quanzhou Medical College, Fujian, China; b State Key Laboratory of Food Science and Resources, International Joint Research Laboratory for Lipid Nutrition and Safety, School of Food Science and Technology, Jiangnan University, Wuxi, China; c School of Agriculture, Food and Ecosystem Sciences, Faculty of Science, University of Melbourne, Melbourne, Australia

**Keywords:** Oral microbiota, periodontal disease, 16S rRNA gene, NHANES, machine learning

## Abstract

**Objective:**

This study aims to characterise the relationship between the oral microbiota and periodontal disease (PD) by leveraging the latest and largest oral microbiota database from the US National Health and Nutrition Examination Survey (NHANES).

**Methods:**

This study represented a secondary analysis of publicly available data from the NHANES 2009–2012 cycle. Within this dataset, subjects with PD and periodontally healthy controls were identified. Oral rinse samples were collected by the original NHANES study, which also performed polymerase chain reaction (PCR) amplification targeting the V4 hypervariable region of the 16S rRNA gene, sequencing, and subsequent construction of amplicon sequence variant (ASV) tables with taxonomic classification using the SILVA reference database. Venn diagram was generated to illustrate the overlap of differentially relative abundant genera identified by the Wilcoxon test, STAMP, and Linear discriminant analysis effect size (LEfSe). These results were integrated to calculate the Microbial Dysbiosis Index (MDI). To reliably distinguish PD, four supervised machine learning (ML) algorithms were employed, SHapley Additive exPlanations (SHAP) was utilised to explain the model.

**Results:**

A Venn diagram identified 19 core genera. Subjects in the case group exhibited a significantly higher MDI compared to controls (*t* = 8.536, *P* < 0.001), with an area under the curve (AUC) of 0.595 (95% confidence interval [CI]: 0.574–0.622). ML models, particularly XGBoost, demonstrated strong predictive performance (AUC: 0.958, 95% CI: 0.950–0.966) for PD classification. SHAP analysis highlighted important microbial taxa, including *Treponema_2* and *Prevotella*.

**Conclusion:**

This study comprehensively investigated the oral microbiota’s association with PD, identifying potential biomarkers for diagnosis and targeted interventions.

## Introduction

Periodontal disease (PD) is a chronic inflammatory disorder triggered by dysbiotic microbial biofilms, characterised by progressive destruction of periodontal tissues [1]. Severe PD, if left untreated, although not fatal, can lead to the total loss of permanent teeth/tooth loss, chewing dysfunction, and nutritional problems. Furthermore, beyond its oral impacts, it has been connected to elevated risks of multiple systemic diseases [2–5].

According to the Global Burden of Disease (GBD), the incidence of periodontitis has increased dramatically over the past 30 years, with the age-standardised prevalence rate rising by 8.44% [6]. An evidence-based review integrating pooled analyses of observational studies found that between 2011 and 2020, periodontitis affected around 62.0% of adults with teeth, with severe periodontitis affecting 23.6% [7]. Periodontitis represents a major public health issue globally. Its widespread occurrence has been shown to substantially increase the disease burden associated with chronic non-communicable conditions.

Pathogenic microorganisms play a crucial role in the progression of PD. These microbial pathogens initiate periodontal inflammation, leading to the progressive destruction of periodontal tissues [8]. In addition to the frequently investigated gut microbiome [9], the oral cavity is also an essential component of this microbiota community, establishing a critical microbial niche hosting 0.5–1 trillion bacterial cells with biodiversity encompassing more than 700 distinct species [8]. Occupying the primary gateway of the digestive system, the oral microbiota plays a pivotal role in regulating nutrient assimilation, biochemical transformations, preventing the invasion and proliferation of pathogens and immunological regulation mechanisms [10, 11]. The oral microbiome’s vital health contributions have driven growing scientific and clinical interest [12].

Extensive research has established that dysbiosis of oral microbial communities contributes etiologically to the inflammatory processes driving periodontitis [13–19]. These contradictory results may be firstly ascribed to the fact that small sample size resulted in inadequate statistical power to detect ecological differences between the groups. However, contradictory findings persist, particularly regarding the ecological mechanisms by which pathobionts, both established and emerging, interact to orchestrate dysbiotic shifts. Periodontitis remains a significant public health concern in the United States of America (US), as highlighted by recent epidemiologic trends and its inclusion among the 42 health priorities in Healthy People 2022 [20]. Despite substantial progress in microbiome research, critical knowledge gaps remain in understanding precisely how changes in species composition disrupt symbiotic host-microbe homeostasis. To address this knowledge gap, the present study seeks to elucidate the relationship between PD and oral microbiome composition by leveraging the largest and most recent nationally representative oral microbiota dataset from the US National Health and Nutrition Examination Survey (NHANES).

## Methods

### Study population

Subjects with PD (Case group) and periodontally healthy controls (Control group) were recruited from the NHANES. The NHANES is a comprehensive, cross-sectional survey employing a stratified, multistage probability sampling design to yield nationally representative estimates of the noninstitutionalised US population. This regular ongoing survey begins with in-home interviews led by trained staff who gather self-reported data on social demographics, health behaviours, and other relevant factors. Following these interviews, participants visit mobile examination centres (MECs) where they undergo standardised physical examinations. At these MECs, trained technicians measure vital statistics such as height and weight, and collect blood and oral rinse samples for laboratory analysis. The NHANES thus combines questionnaire data with clinical assessments and biological measurements to provide a detailed portrait of health and nutritional status among community-dwelling residents across the nation.

Our analysis utilised cross-sectional data (2009–2012 cycles) from participants aged 30–69 years, with oral rinse samples collected during the study period. This data can be accessed on the website at https://wwwn.cdc.gov/Nchs/Nhanes/omp/.

This study, approved by the National Center for Health Statistics (NCHS) Ethics Review Board (NCHS IRB/ERB Protocol Number: #2011–17), involved obtaining written informed consent from all NHANES participants. It received an exemption from the Yale University Institutional Review Board due to the public availability and deidentification of NHANES data.

### Definition of covariates

Demographic variables, including age, sex, self-reported race, marital status, educational level, alcohol intake, smoking status, physical activity, history of cardiovascular disease (CVD), history of cancer, hypertension, and diabetes were ascertained by an in-person questionnaire. Poverty status was determined using the poverty-income ratio (PIR), calculated as household income divided by the U.S. Census Bureau’s poverty threshold adjusted annually based on household size and location. Smoking categories were classified as: never smoker (applies to those reporting no lifetime cigarette use or consuming fewer than 100 cigarettes); former smoker (includes individuals with prior smoking history [≥ 100 cigarettes] who currently abstain from tobacco/nicotine for ≥ 5 days); and current smoker (refers to active tobacco/nicotine users within the past 5 days). Alcohol intake was categorised as: never drinkers (lifetime consumption < 12 drinks); former drinkers (≥ 12 lifetime drinks but abstained for ≥ 12 months); light drinkers (0–0.5 daily drinks); moderate drinkers (0.5–1.5 for women or 0.5–2.5 for men daily drinks); and heavy drinkers (≥ 1.5 for women or ≥ 2.5 for men daily drinks). Total weekly moderate-intensity activity time (TWMT) was defined as the sum of original moderate-intensity activity duration and vigorous-intensity activity converted to moderate-equivalent time during a typical week [4]. Physical activity levels were categorised into three groups: active (≥ 150 min TWMT); insufficient (10–149 min TWMT); and inactive (no participation or < 10 min TWMT).

### Periodontal disease

Self-reported health status measures are widely used in population-based studies to assess systemic diseases and health-related conditions. Previous research demonstrates good validity for self-reported oral health questions and self-reported PD in large populations [21–23]. Moreover, self-reported periodontal screening questions perform adequately in identifying clinically defined PD [24]. Self-reported oral health is also an indicator with high reliability and validity, comparable to the results of an oral examination performed by a dentist [25]. The evaluation of PD used the questions: ‘Do you think you might have gum disease?’ We treated this variable as a binary outcome: ‘Do you think you might have gum disease?’ (yes/no). A positive response to this diagnostic inquiry indicates a diagnosis of PD. Gum disease is clinically characterised by gingival inflammation (swelling), tissue recession, localised tenderness or infection, and tooth mobility.

### 16S rRNA sequence processing and analysis

Oral rinse samples were collected by rinsing with a mouthwash for 5s and then gargling three times for 5s; DNA was extracted using methods described in detail previously [26]. Details of all bioinformatics procedures and included code were available on the NHANES website [27]. The hypervariable V4 region of the 16S rRNA gene was amplified via polymerase chain reaction (PCR) and subjected to sequencing analysis to establish the microbiota of the samples. The sequencing data were processed using QIIME 2 and DADA2 software (version 1.2.1) to generate amplicon sequence variant (ASV) tables with taxonomy based on the SILVA version 123 database [28].

Alpha diversity quantifies the richness and evenness of microbial communities within individual samples, with established ecological metrics including observed ASVs, Faith’s Phylogenetic Diversity, the Shannon-Wiener Index, and the Inverse Simpson Index. These indices were calculated using rarefaction thresholds ranging from 2,000 to 10,000 sequences.

Beta diversity, a metric quantifying differences in microbial communities across samples, is operationalised in these datasets through pairwise dissimilarity indices. A distance matrix included unweighted UniFrac, weighted UniFrac, and Bray-Curtis dissimilarity. Principal Coordinates Analysis (PCoA) was used to visualise the differences between the two groups. To assess the statistical significance of the observed group differences visualised via PCoA, we employed the Adonis permutation-based test. This method, formally known as Permutational Multivariate Analysis of Variance (PERMANOVA), partitions sums of squares within multivariate datasets to identify significant differences among sample groups.

### Genus level differences analysis

ASVs with relative abundance lower than 0.02% and present in less than 20.0% of samples were excluded. Initial comparisons of the differential relative abundance of genera between two groups were tested by the Mann–Whitney Wilcoxon signed rank test, and *P*-value was corrected as false discovery rate (FDR) with the Benjamini–Hochberg (B–H) method. We used STAMP software (version 2.1.3) to identify differentially abundant taxa, employing Welch’s t-test with B–H FDR correction for multiple testing [23]. Linear discriminant analysis effect size (LEfSe) was utilised to filter the discriminating taxa across groups with the linear discriminant analysis (LDA) ≥ 2 [29]. A Venn diagram was constructed of the differentially abundant genera identified between the Wilcoxon test, LEfSe, and STAMP results. The microbiota dysbiosis index (MDI) quantifies the degree of microbial imbalance. This index is calculated per sample using the formula: log ([total abundance of bacterial taxa increased in Case group] / [total abundance of bacterial taxa decreased in Case group]). The abundances used in this calculation were based on the differentially abundant genera identified. The MDI exhibited a strong positive correlation with clinical disease severity [30].

### Machine learning approaches

To determine a set of microbial features that could reliably distinguish PD, four widely used and high-performing supervised ML algorithms were employed, including eXtreme Gradient Boosting (XGBoost), K-Nearest Neighbour (KNN), Support Vector Machine (SVM), and Random Forest (RF). The data were randomly split into training and validation sets at a 7:3 ratio. Optimal hyperparameters were determined using a grid search with 5-fold cross-validation on the training set, by ‘auto_tuner’ function, to adjust parameters to improve the performance of the ML models. The discriminative performance of various ML models was assessed through the construction of receiver operating characteristic (ROC) curves and calculation of the corresponding area under the curve (AUC) values [35]. In addition, a comprehensive set of evaluation metrics was employed, including sensitivity, specificity, positive predictive value (PPV), negative predictive value (NPV), recall, precision and confusion matrix. The robustness and clinical applicability of the ML models were validated by employing calibration curve analysis and decision curve analysis (DCA). We quantified feature contributions in predictive models using SHapley Additive exPlanations (SHAP) values, which reveal how variables influence predictions.

### Statistical analysis

We first performed data cleaning, no missing data were present for demographic variables, participants who were pregnant or lactating were excluded from the study. The final dataset comprised of participants with complete information on all relevant variables.

The propensity score matching (PSM) method was employed to mitigate the impact of confounding factors, ensuring a more accurate comparison between the two groups. Utilising a caliper value of 0.05, the nearest neighbour matching method was employed for 1:3 matching.

All statistical analyses were evaluated using R version 4.3.1. PSM was performed with the ‘MatchIt’ package. Machile learning models were carried out through ‘mlr3verse’ package. All *P*-values were 2-sided, and *P* < 0.05 was considered statistically significant.

## Results

### Demographics of the study population

**Figure 1:**
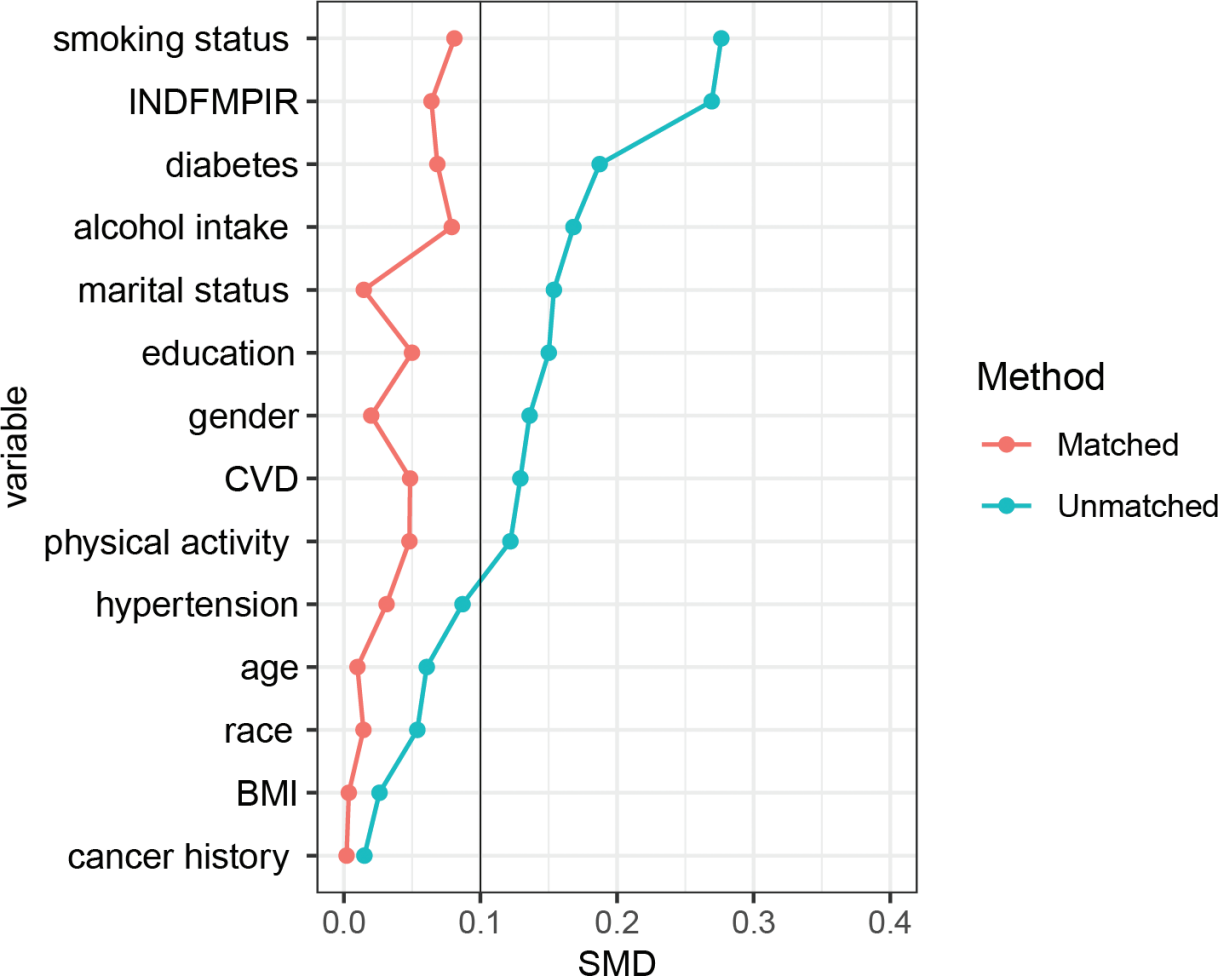
Standardised mean differences (SMD) for baseline characteristics across methods.

After initial data cleaning, 772 individuals with PD and 3021 periodontally healthy control were included. Table 1 and Figure 1 present the standardised mean differences (SMD) for age, sex, race, marital status, education level, alcohol consumption, smoking status, physical activity, PIR, history of CVD, history of cancer, hypertension, and diabetes. However, significant differences between the two groups were observed for smoking status, PIR, diabetes, and alcohol consumption. To controlling for potential confounding variables for further microbial analysis, we employed the PSM method to ensure balanced comparability between the groups. The selected participants (Case group: *n* = 760; Control group: *n* = 2,056) were well-balanced on sociodemographic characteristics (all *P* > 0.05; all SMD < 0.1), as detailed in Table 1 and Figure 1.

**Table 1:** Sociodemographic characteristics between Case and Control groups.

Variables	Overall data				Propensity 1:3 Matching			
	Case	Control	*P*	SMD	Case	Control	*P*	SMD
	*n* = 772	*n* = 3,021			*n* = 760	*n* = 2,056		
Age (mean, SD)	49.13 (11.18)	48.44 (11.35)	0.134	0.061	49.09 (11.20)	48.97 (11.42)	0.815	0.01
Gender (%)			0.001	0.136			0.668	0.02
Female	342 (44.30)	1,543 (51.08)			341 (44.87)	943 (45.87)		
Male	430 (55.70)	1,478 (48.92)			419 (55.13)	1,113 (54.13)		
Race (%)			0.411	0.054			0.944	0.014
Hispanic	226 (29.27)	857 (28.37)			223 (29.34)	613 (29.82)		
Non-Hispanic Black	205 (26.55)	751 (24.86)			199 (26.18)	543 (26.41)		
Non-Hispanic White	341 (44.17)	1,413 (46.77)			338 (44.47)	900 (43.77)		
BMI (mean, SD)	29.72 (6.36)	29.56 (6.04)	0.511	0.026	29.67 (6.36)	29.70 (6.08)	0.931	0.004
Marital status (%)			< 0.001	0.154			0.764	0.015
Married/live with other	449 (58.16)	1,982 (65.61)			447 (58.82)	1,224 (59.53)		
Others	323 (41.84)	1,039 (34.39)			313 (41.18)	832 (40.47)		
Education level (%)			0.001	0.15			0.502	0.05
Greater than high school	372 (48.19)	1,681 (55.64)			367 (48.29)	1,044 (50.78)		
High school diploma or GED	192 (24.87)	653 (21.62)			188 (24.74)	482 (23.44)		
Less than high school	208 (26.94)	687 (22.74)			205 (26.97)	530 (25.78)		
PIR (mean, SD)	2.31 (1.60)	2.75 (1.70)	< 0.001	0.269	2.33 (1.60)	2.44 (1.65)	0.134	0.064
Alcohol intake (%)			0.001	0.168			0.467	0.079
Former drinker	104 (13.47)	429 (14.20)			103 (13.55)	307 (14.93)		
Heavier drinker	84 (10.88)	200 (6.62)			80 (10.53)	174 (8.46)		
Light drinker	328 (42.49)	1,362 (45.08)			327 (43.03)	896 (43.58)		
Moderate drinker	182 (23.58)	673 (22.28)			176 (23.16)	468 (22.76)		
Never drinker	74 (9.59)	357 (11.82)			74 (9.74)	211 (10.26)		
Smoking status (%)			< 0.001	0.276			0.158	0.081
Current smoker	266 (34.46)	685 (22.67)			254 (33.42)	610 (29.67)		
Former smoker	156 (20.21)	616 (20.39)			156 (20.53)	442 (21.50)		
Never smoker	350 (45.34)	1,720 (56.93)			350 (46.05)	1,004 (48.83)		
Physical activity (%)			0.011	0.122			0.529	0.048
Active	228 (29.53)	1,052 (34.82)			225 (29.61)	646 (31.42)		
Inactive	426 (55.18)	1,497 (49.55)			419 (55.13)	1,085 (52.77)		
Insufficient active	118 (15.28)	472 (15.62)			116 (15.26)	325 (15.81)		
Cancer history (%)			0.772	0.015			1	0.002
No	721 (93.39)	2,810 (93.02)			709 (93.29)	1,917 (93.24)		
Yes	51 (6.61)	211 (6.98)			51 (6.71)	139 (6.76)		
CVD (%)			0.001	0.129			0.278	0.049
Yes	75 (9.72)	188 (6.22)			69 (9.08)	159 (7.73)		
No	697 (90.28)	2,833 (93.78)			691 (90.92)	1,897 (92.27)		
Hypertension (%)			0.033	0.087			0.488	0.031
No	480 (62.18)	2,004 (66.34)			475 (62.50)	1,316 (64.01)		
Yes	292 (37.82)	1,017 (33.66)			285 (37.50)	740 (35.99)		
Diabetes (%)			< 0.001	0.187			0.115	0.068
No	644 (83.42)	2,712 (89.77)			640 (84.21)	1,781 (86.62)		
Yes	128 (16.58)	309 (10.23)			120 (15.79)	275 (13.38)		

SD: standard deviation; SMD: standardised mean differences; PIR: poverty-income ratio; CVD: cardiovascular disease.

### Microbiota profiling in case and control groups

Rarefaction curves typically exhibit a steep initial rise as new taxa are detected, gradually approaching an asymptote as sequencing depth increases. In this study, the genus-level curve reached saturation at 10,000 reads, indicating that further sequencing yields diminishing returns for novel species discovery.

**Figure 2:**
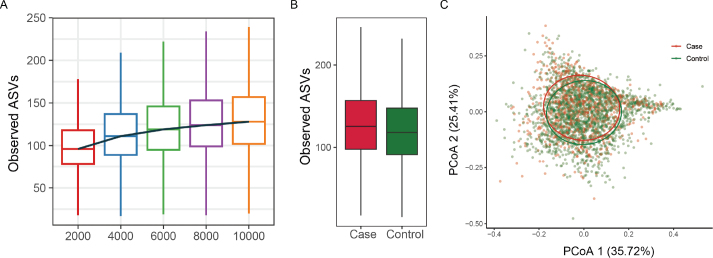
(A) Rarefaction curve showing the observed amplicon sequence variants (ASVs) across different sequencing depths. (B) Boxplot comparing the alpha diversity (observed ASVs) between Case and Control groups. (C) Principal Coordinate Analysis (PCoA) plot illustrating beta diversity, with PCoA 1 (35.72%) and PCoA 2 (25.41%) explaining the variance between Case and control groups.

We focused on the observed number of ASVs, it’s alpha-diversity metrics showed Case group have a higher level of oral microbial richness and diversity in genus level, Figure 2B (*P* < 0.01). Another rarefaction curves and alpha diversity results including faith’s Phylogenetic index, Shannon-Weiner index and Inverse Simpson index were shown in Supplementary Figure 1 and Supplementary Figure 2.

Analysis of beta diversity, the Adonis test results based on weighted and unweighted UniFrac distances, and Bray-Curtis distance measures showed that the oral bacterial community composition was significantly different (All *P* < 0.001). In Figure 2C, for the weighted UniFrac distances index, the PCoA 1 accounts for 35.72% of the variance and PCoA 2 accounts for 25.41%. The results for Faith’s Phylogenetic Diversity Index, Inverse-Simpson Index, and Shannon-Weiner Index are presented in Supplementary Figure 3.

### Differential abundance of taxa between two groups

**Figure 3:**
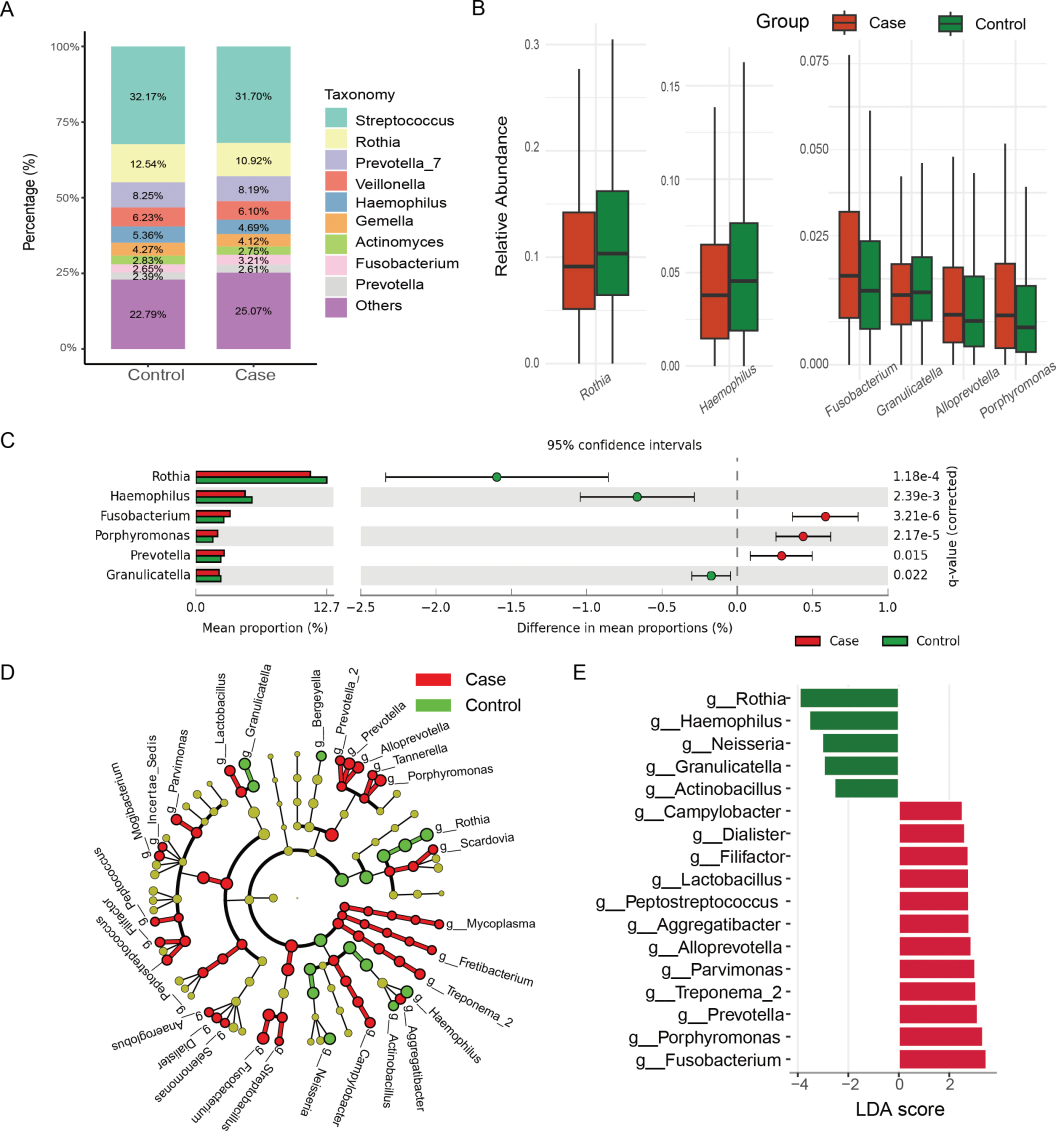
(A) The top 10 genera with the highest relative abundance at the genus level between the two groups (B) Differences relative abundance in predominant bacterial taxon between Case and Control groups. (C) Differences in mean proportions of the bacterial taxa between Case and Control groups from STAMP result. (D) Cladogram depicting the distribution of differentially abundant microbiota taxa in a phylogenetic tree, with red for Case-enriched and green for Control-enriched taxa. (E) Linear Discriminant Analysis (LDA) scores revealed significant bacterial disparities through LEfSe (LDA effect size) analysis.

Following rigorous data preprocessing and quality control, across all the samples, a total of 9 bacterial phyla and 84 different genera were found. *Streptococcus, Rothia*, *Prevotella_7*, *Veillonella*, *Haemophilus*, *Gemella*, *Actinomyces*, *Fusobacterium,* and *Prevotella* were the top dominant bacteria of oral microbiota in both groups (Figure 3A). *Streptococcus* comprised up to over 30%, was among the most abundant genera, but were no difference between two groups.

We searched for microbial taxa with the potential to serve as biomarkers distinguishing two groups. The most frequently difference detected taxon in each group were shown in Figure 3B (only those above 1% in relative abundance were listed), the relative abundance of *Rothia, Haemophilus* and *Granulicatella* in the PD group were significantly higher. Others differentiated taxon are showed in Supplementary Figure 4.

The STAMP software results detailed showed the difference in mean proportions, partly consistent with above Wilcoxon test (see Figure 3C and Supplementary Figure 5). The LEfSe analysis identified specific microbiota compositions between the two groups, LDA > 2.5 in genus level are showed in Figure 3E, all LDA value are shown in Supplementary Table 3. The cladogram illustrated the disparities in the phylogenetic tree (Figure 3D), such as the higher proportions of five genus (genus *Alloprevotella*, genus *Tannerella*, genus *Porphyromonas*, genus *Prevotella*, genus *Prevotella_2*) associated with the PD were gathered in class *Bacteroidia*, the differential abundance of the genus *Scardovia* and genus *Rothia* between the two groups was traced to a higher taxonomic level, specifically to their parent order, *Bifidobacteriales*. Notably, certain taxa associated with both disease and health fall within the same Phylum (e.g. *Proteobacteria* and *Actinobacteria*). The detail difference taxon levels can be seen in Supplementary Table 2.

### Characterised microbial taxa distinguish two groups

**Figure 4:**
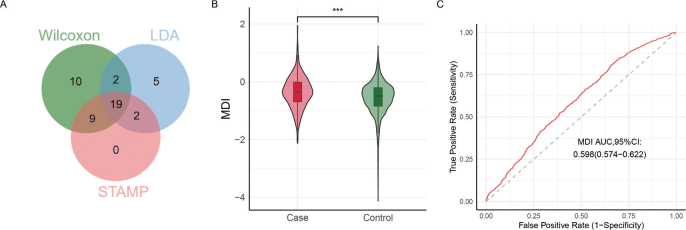
(A) Venn diagram illustrating the number of shared and unique taxa at the genus level across different analysis methods. (B) The comparison of Microbial Dysbiosis Index (MDI) between two groups. Top lines show the *p*-values of Kruskal-Wallis-U Mann Whitney tests for the comparisons among groups. (C) Receiver Operating Characteristic (ROC) curve analysis evaluating the discriminatory potential of the MDI index.

Instead of relying solely on a single taxon, it is essential to take the overall microbial composition into account. And to improve the reliability of the results to distinguish, a Venn diagram was constructed and displayed the core genera among the above three approaches, including *Anaeroglobus*, *Campylobacter*, *Dialister*, *Filifactor*, *Fretibacterium*, *Fusobacterium*, *Granulicatella*, *Haemophilus*, *Mogibacterium*, *Mycoplasma*, *Parvimonas*, *Peptococcus*, *Peptostreptococcus*, *Porphyromonas*, *Prevotella_2*, *Rothia*, *Selenomonas*, *Tannerella*, *Treponema_2*. The 19 differentially abundant genera might be the potential biomarker and were further combined to estimate the MDI. As presented in Figure 4, compared with their control counterparts, subjects in the Case group had a higher MDI (*t* = 8.536, *P* < 0.001), and AUC indicated that the model has a certain level of classification ability (AUC, 95% confidence interval [CI]: 0.595, 0.574–0.622). Machine learning (ML) algorithm can provide a more powerful classification effect. Four ML classifiers were carried out.

**Figure 5:**
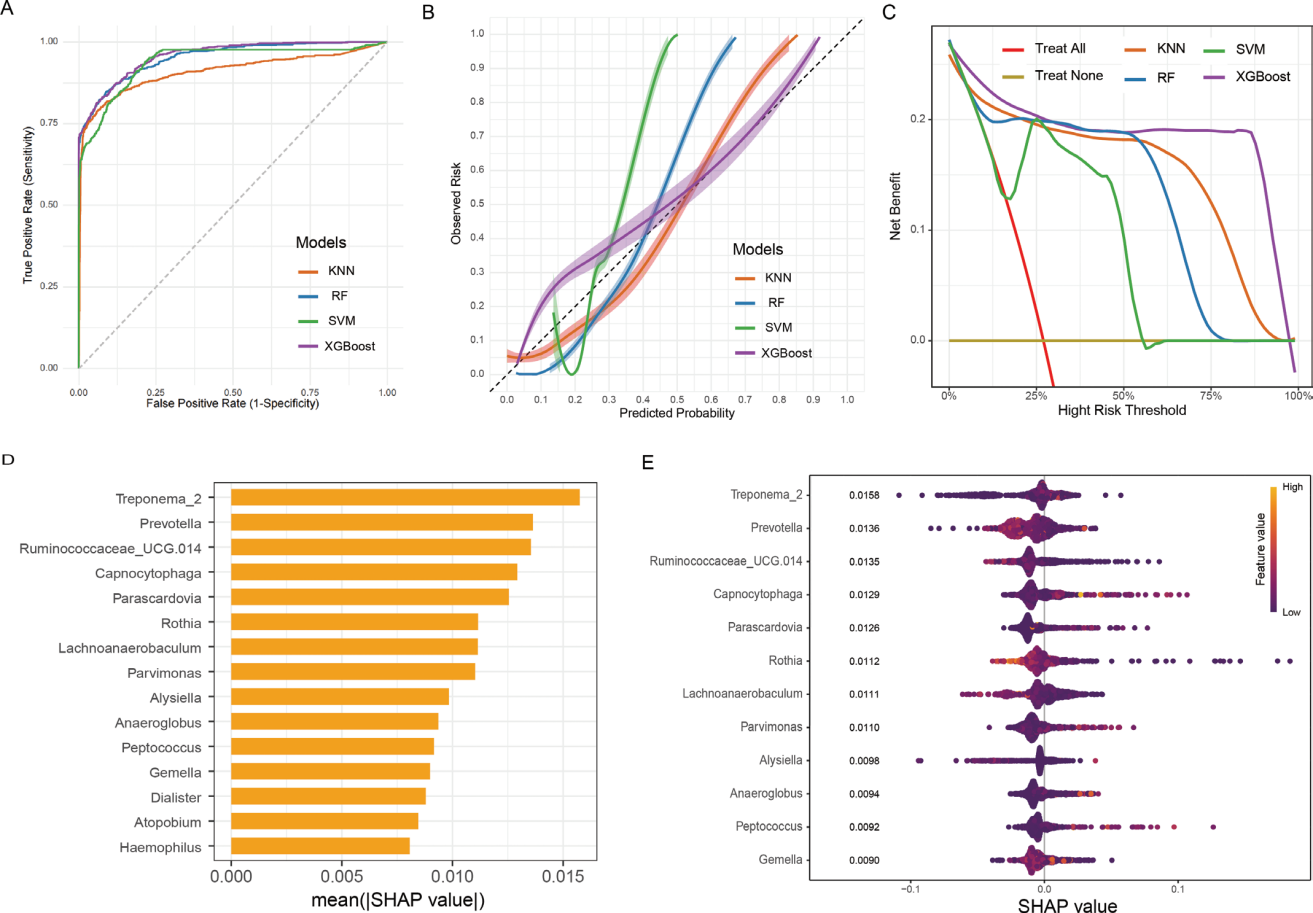
(A) Receiver Operating Characteristic (ROC) curve analysis of machine learning algorithms for prediction of Periodontal disease (PD). (B) Calibration plots for predicting PD with various models and (C) Decision curve analysis (DCA) for each model. (D) Importance Mean SHAP Value results from XGBoost model and (E) SHAP summary plot for the XGBoost model. Abbreviations: PD: Periodontal Disease; XGBoost: extreme gradient boosting; RF: random forest; KNN: K-nearest neighbour; SVM: support vector machine; ROC: receiver operating characteristic; DCA: decision curve analysis.

The small observed performance differences (training vs. test AUC: XGBoost = 0.042, RF = 0.011, SVM = 0.017, KNN = 0.086) indicate that these models generalised well to the unseen test data without severe overfitting. We evaluated and synthesised the performance outcomes of various ML classification models (Table 2 and Figure 5A). Among these, the XGBoost model demonstrated superior predictive performance, with an AUC of 0.958 (95% CI: 0.950–0.966). It also achieved an accuracy of 0.919, a sensitivity of 0.724, and a specificity of 0.991.

**Table 2:** Predictive performance of the four machine learning algorithms.

Learner	AUC, 95% CI	Sensitivity	Specificity	Precision	Recall	NPV	PPV	Accuracy	Confusion Matrix	
RF	0.952 (0.944–0.961)	0.705	0.997	0.987	0.705	0.901	0.987	0.918	536	7
									224	2,049
XGBoost	0.958 (0.950–0.966)	0.724	0.991	0.968	0.724	0.907	0.968	0.919	550	18
									210	2,038
SVM	0.939 (0.927–0.950)	0.464	1.000	0.997	0.464	0.835	0.997	0.855	353	1
									407	2,055
KNN	0.909 (0.894–0.925)	0.741	0.974	0.914	0.741	0.91	0.914	0.911	562	53
									197	2,003

AUC: area under the curve; CI: confidence interval; PPV: positive predictive value; NPV: negative predictive value; RF: random forest; XGBoost: extreme gradient boosting; SVM: support vector machine; KNN: K-nearest neighbour.

In addition, an accuracy assessment of the models was performed through calibration plot analysis (Figure 5B), and the XGBoost model demonstrated strong predictive accuracy. The DCA serves as an effective tool for evaluating the clinical applicability of disease diagnostic models. As illustrated in Figure 3C, the DCA results demonstrated that the XGBoost algorithm achieved superior clinical performance compared to other models.

To address clinicians’ challenges in accepting predictive models with interpretability limitations, we utilised SHAP to elucidate the models through quantifying feature contributions. SHAP value analysis was performed on the XGBoost algorithm to identify key microbial species variations across groups. Key features ranked by SHAP value importance were *Treponema_2*, *Prevotella*, *Ruminococcaceae_UCG.014*, *Capnocytophaga*, *Parascardovia*, *Rothia*, *Lachnoanaerobaculum*, *Parvimonas*, *Alysiella*, *Anaeroglobus*, *Peptococcus*, and *Gemella* (Figure 5D). The beeswarm plot (Figure 5E) displays a consistent trend with the above results. Specifically, when the feature value and the SHAP value both decline, it implied a negative relationship with the PD. For example *Rothia*, had the greatest relative abundance among the different taxa in both groups, was more in the Control group; this corresponds to lower feature values and increased SHAP values.

## Discussion

This large-scale NHANES study examined the link between the oral microbiota and PD. PSM was employed to control for potential confounding variables. Initially, microbiota profiling was conducted, encompassing rarefaction curve, alpha diversity, and beta diversity. In 84 genera, *Rothia*, *Haemophilus*, and *Granulicatella* were more abundant in the PD group. A Venn diagram pinpointed 19 genera, which formed the MDI that was elevated in PD cases. XGBoost accurately differentiated PD from healthy controls, with SHAP analysis identifying features showing both significant variation and strong correlation with disease status.

PD has been shown to be a polymicrobial disruption of host homeostasis [31]. We found that PD group exhibited significantly higher alpha diversity compared to healthy controls. The formation of deep periodontal pockets in periodontitis may provide novel ecological niches, potentially explaining the proliferation of disease associated microbial species in these habitats [15]. Several previous studies have evaluated the association between the taxonomic composition of the microbiota and PD, using 16S rRNA gene profiling [13–19]. The classic study conducted by Socransky et al. established significant correlations between PD and specific bacterial pathogens, notably identifying *Porphyromonas*, *Treponema*, and *Tannerella* species that were collectively designated as the red complex [32]. Shi et al. found that *Porphyromonas*, *Treponema*, *Peptostreptococcus*, *Filifactor*, *Mycoplasma* were significantly more abundant in the diseased state [18]. *Peptostreptococcus*, a recognised member of the orange complex [32], has been extensively documented as a pathogenic contributor to periodontitis, with multiple studies confirming this association [13, 15, 17]. *Fusobacterium* was demonstrated a relatively high abundance in both study populations, with particularly pronounced presence in periodontitis samples. Previous research has consistently shown that this genus exhibits more extensive microbial partnerships than any other genus within the oral microbiota [33]. This characteristic strongly suggests its crucial pathological role, potentially functioning as a pivotal bridge species that facilitates the colonisation of more pathogenic microorganisms in subgingival biofilms [34]. Furthermore, the control group exhibited elevated levels of bacterial genera not typically linked to PD. Specifically, saliva-enriched taxa including *Streptococcus*, *Neisseria,* and *Rothia* were predominantly observed in this healthy population [35]. Our findings demonstrate consistent characteristic patterns of these microorganisms in PD across different oral sample types, such as rinses and subgingival plaque, aligning with previous studies [13–15, 35, 36].

Multiple studies have reported elevated levels of *Treponema_2* species in saliva, periodontal pockets, and subgingival plaque samples from patients with periodontitis [37–39]. Although specific informal designations were identified that corresponded to pathogenic strains within the genus *Treponema*, it encompasses over 20 species, some of which are recognised pathogens. One such example is *Treponema denticola*, a member of the bacterial consortium previously designated as the ‘red complex’. This pathogen invades and destroys periodontal tissue and promotes immunodestructive host responses [40]. Unique virulence mechanisms of *T. denticola* include the ability to induce and degrade cytokines and to inhibit the migration of fibroblasts and neutrophils. Consequently, its effects on neutrophils may lead to incomplete clearance of periodontopathogenic bacteria, including *T. denticola* itself, while its inhibitory effects on fibroblasts could delay wound healing [40, 41]. *Prevotella*, strongly associated with periodontitis and grouped within the ‘orange complex’ [42], possess several virulence factors, such as protease production, capsule formation, antibiotic resistance genes, biofilm capacity, and secretion systems that evade host defenses, which aid in colonisation, mediate interactions with other bacteria, and contribute significantly to PD progression [43]. Given the established pathogenicity of *Treponema* species (e.g. *T. denticola*) and *Prevotella* in PD, the high diagnostic accuracy (AUC = 0.958) of the XGBoost model developed in this study underscores the power of ML to integrate complex microbial signatures. SHAP analysis further identified *Treponema_2* and *Prevotella* as key predictive taxa. Critically, these genera not only served as high-value diagnostic biomarkers but also represented mechanistically grounded targets for therapeutic intervention.

This work confirmed and expanded previous findings by demonstrating that PD is associated with a complex microbial community rather than a single pathogen, while identifying specific species that are more prevalent in disease conditions, and contributing to our understanding of putative pathogenic species. However, certain microbial species linked to diseases are detectable in healthy individuals but proliferate under pathological conditions [44]. Of particular scientific interest are pathogens exclusively identified in diseased states, as their unique presence strongly correlates with disease mechanisms. Although health-promoting microbiota show reduced abundance during illness without complete elimination [15], this suggests that probiotic interventions targeting beneficial strains may prove inadequate unless concurrent strategies specifically targeting pathogenic organisms are implemented. Crucially, LEfSe analysis and the phylogenetic cladogram revealed that PD-associated genera (*Alloprevotella*, *Tannerella*, *Porphyromonas*, *Prevotella*, *Prevotella_2*) were evolutionarily clustered within the class *Bacteroidia*. This suggests that interventions targeting metabolic pathways or conserved molecular features shared across this taxonomic class, including designing *Bacteroidia*-specific inhibitors, could offer an effective broad-spectrum therapeutic strategy against these pathogenic clusters.

Recent research has proposed the dysbiosis hypothesis [45, 46], which delineates a reciprocal relationship between oral microbiota imbalance and chronic inflammation. These pathological processes engage in a mutually reinforcing cycle, wherein bacteria either directly inflict damage or elicit detrimental inflammatory host responses, thereby synergistically driving the progression of periodontitis [47]. *Porphyromonas* species (e.g. *Porphyromonas gingivalis*) and *Treponema* species (e.g. *Treponema denticola*) are well-known periodontal pathogens. They contribute to tissue destruction, notably through the secretion of proteases such as the gingipains produced by *P. gingivalis*, and simultaneously provoke chronic inflammatory responses in the host [48–50]. The flagellar structures of *Selenomonas* penetrate the epithelial barrier and activate the NLRP3 inflammasome, triggering an excessive inflammatory response through caspase-1-mediated maturation of IL-1β and IL-18 [50]. *Peptostreptococcus* induces NLRP3 inflammasome activation in macrophages, this triggers pyroptosis that depends on gasdermin D cleavage and pore formation, resulting in the subsequent release of interleukin-1β [51].

Our study has several strengths, notably the employment of an extensive dataset with national representativeness, meticulous control of confounding variables through thorough statistical measures, and systematic analysis of the oral microbiota. However, several limitations need to be recognised. First, the cross-sectional design of NHANES precludes causal inference, making it challenging to determine the temporal relationship between PD and oral dysbiosis. We cannot ascertain whether PD precedes or follows microbial alterations, or if declining health status simultaneously affects both PD and oral microbial communities. Furthermore, PD was assessed solely using a single binary self-reported question, potentially limiting the interpretation of the results. Nevertheless, as a large-scale investigation linking the oral microbiota to PD, our findings provide valuable insights for future longitudinal studies. However, external validation using independent cohorts is necessary to rigorously assess the portability of our microbial signature and XGBoost model. Second, while the oral microbiome exhibits considerable resilience compared to gut microbiota, its composition can still be influenced by multiple factors, including dietary intake, host immunity, medication use, and systemic diseases. Although our study controlled for major confounders, residual confounding from unmeasured factors cannot be completely ruled out. Third, several methodological limitations warrant consideration. While the analysis of oral fluid samples offers a non-invasive method well-suited for large-scale epidemiological studies, it may not fully capture the complex microbial ecology linked to PD pathogenesis. In contrast, subgingival plaque samples provide a more accurate representation of microenvironmental changes critical to PD development and progression [52]. Finally, although species-level analysis is technically feasible with methods like 16S rRNA gene sequencing and whole genome analysis, our secondary analysis of the processed NHANES data remained constrained, resulting in a taxonomic resolution limited to the genus level. This resolution constraint became particularly problematic when attempting species-level identification of *Porphyromonas gingivalis*, a keystone periodontopathogen whose strain-specific virulence factors critically influence its pathogenic potential.

## Conclusion

Overall, this study represented a comprehensive, large-scale investigation into the association between the oral microbiota and PD. We employed multiple analytical approaches to conduct differential analysis, and furthermore, utilised advanced ML techniques to effectively classify samples and interpret the critical contributors to PD prediction. Future research should validate these results and explore causal relationships to inform clinical applications.

### Human ethics and consent to participate declarations

This study, approved by the NCHS Ethics Review Board (NCHS IRB/ERB Protocol Number: #2011–17), involved obtaining written informed consent from all NHANES participants. It received an exemption from the Yale University Institutional Review Board due to the public availability and deidentification of NHANES data.

## Supplementary Material





## Data Availability

The datasets analysed in this study are publicly available on the Centers for Disease Control and Prevention (CDC) National Health and Nutrition Examination Survey (NHANES) website: https://wwwn.cdc.gov/Nchs/Nhanes/omp/.
