# Supplementary material for: Characterising oral microbial signatures for periodontal disease in the NHANES population
Source: Acta Odontol Scand. 2026 Mar 30;85:45662. doi: 10.2340/aos.v85.45662 (PMC13058696; doi:10.2340/aos.v85.45662)
Supplement: Supplementary file 1 [file AOS-85-45662-s1.pdf]

Supplementary material has been published as submitted. It has not been copyedited or typeset by Acta Odontologica Scandinavica.

Supplementary Table 1: Bacterial taxonomic classification from SILVA version 123 database

Taxonomy
k__Bacteria|p__Actinobacteria|c__Actinobacteria|o__Actinomycetales|f__Actinomycetaceae|g__Actinomyces
k__Bacteria|p__Actinobacteria|c__Actinobacteria|o__Bifidobacteriales|f__Bifidobacteriaceae|g__Alloscardovia
k__Bacteria|p__Actinobacteria|c__Actinobacteria|o__Bifidobacteriales|f__Bifidobacteriaceae|g__Bifidobacterium
k__Bacteria|p__Actinobacteria|c__Actinobacteria|o__Bifidobacteriales|f__Bifidobacteriaceae|g__Parascardovia
k__Bacteria|p__Actinobacteria|c__Actinobacteria|o__Bifidobacteriales|f__Bifidobacteriaceae|g__Scardovia
k__Bacteria|p__Actinobacteria|c__Actinobacteria|o__Corynebacteriales|f__Corynebacteriaceae|g__Corynebacterium
k__Bacteria|p__Actinobacteria|c__Actinobacteria|o__Micrococcales|f__Micrococcaceae|g__Rothia
k__Bacteria|p__Actinobacteria|c__Coriobacteriia|o__Coriobacteriales|f__Coriobacteriaceae|g__Atopobium
k__Bacteria|p__Actinobacteria|c__Coriobacteriia|o__Coriobacteriales|f__Coriobacteriaceae|g__Cryptobacterium
k__Bacteria|p__Actinobacteria|c__Coriobacteriia|o__Coriobacteriales|f__Coriobacteriaceae|g__Olsenella
k__Bacteria|p__Bacteroidetes|c__Bacteroidia|o__Bacteroidales|f__Bacteroidaceae|g__Bacteroides
k__Bacteria|p__Bacteroidetes|c__Bacteroidia|o__Bacteroidales|f__Bacteroidales_Incertae_Sedis|g__Phocaeicola
k__Bacteria|p__Bacteroidetes|c__Bacteroidia|o__Bacteroidales|f__Porphyromonadaceae|g__Porphyromonas
k__Bacteria|p__Bacteroidetes|c__Bacteroidia|o__Bacteroidales|f__Porphyromonadaceae|g__Tannerella
k__Bacteria|p__Bacteroidetes|c__Bacteroidia|o__Bacteroidales|f__Prevotellaceae|g__Alloprevotella
k__Bacteria|p__Bacteroidetes|c__Bacteroidia|o__Bacteroidales|f__Prevotellaceae|g__Prevotella
k__Bacteria|p__Bacteroidetes|c__Bacteroidia|o__Bacteroidales|f__Prevotellaceae|g__Prevotella_2
k__Bacteria|p__Bacteroidetes|c__Bacteroidia|o__Bacteroidales|f__Prevotellaceae|g__Prevotella_6
k__Bacteria|p__Bacteroidetes|c__Bacteroidia|o__Bacteroidales|f__Prevotellaceae|g__Prevotella_7
k__Bacteria|p__Bacteroidetes|c__Flavobacteriia|o__Flavobacteriales|f__Flavobacteriaceae|g__Bergeyella
k__Bacteria|p__Bacteroidetes|c__Flavobacteriia|o__Flavobacteriales|f__Flavobacteriaceae|g__Capnocytophaga
k__Bacteria|p__Bacteroidetes|c__Flavobacteriia|o__Flavobacteriales|f__Flavobacteriaceae|g__Cloacibacterium
k__Bacteria|p__Firmicutes|c__Bacilli|o__Bacillales|f__Family_XI|g__Gemella
k__Bacteria|p__Firmicutes|c__Bacilli|o__Bacillales|f__Staphylococcaceae|g__Staphylococcus
k__Bacteria|p__Firmicutes|c__Bacilli|o__Lactobacillales|f__Aerococcaceae|g__Abiotrophia
k__Bacteria|p__Firmicutes|c__Bacilli|o__Lactobacillales|f__Carnobacteriaceae|g__Granulicatella
k__Bacteria|p__Firmicutes|c__Bacilli|o__Lactobacillales|f__Lactobacillaceae|g__Lactobacillus
k__Bacteria|p__Firmicutes|c__Bacilli|o__Lactobacillales|f__Streptococcaceae|g__Streptococcus
k__Bacteria|p__Firmicutes|c__Clostridia|o__Clostridiales|f__Clostridiales_vadinBB60_group
k__Bacteria|p__Firmicutes|c__Clostridia|o__Clostridiales|f__Defluviitaleaceae|g__Defluviitaleaceae_UCG-011
k__Bacteria|p__Firmicutes|c__Clostridia|o__Clostridiales|f__Eubacteriaceae|g__Pseudoramibacter
k__Bacteria|p__Firmicutes|c__Clostridia|o__Clostridiales|f__Family_XI|g__Parvimonas
k__Bacteria|p__Firmicutes|c__Clostridia|o__Clostridiales|f__Family_XIII|g__Family_XIII_UCG-001
k__Bacteria|p__Firmicutes|c__Clostridia|o__Clostridiales|f__Family_XIII|g__Incertae_Sedis
k__Bacteria|p__Firmicutes|c__Clostridia|o__Clostridiales|f__Family_XIII|g__Mogibacterium
k__Bacteria|p__Firmicutes|c__Clostridia|o__Clostridiales|f__Family_XIII|g__[Eubacterium]_brachy_group
k__Bacteria|p__Firmicutes|c__Clostridia|o__Clostridiales|f__Family_XIII|g__[Eubacterium]_nodatum_group
k__Bacteria|p__Firmicutes|c__Clostridia|o__Clostridiales|f__Lachnospiraceae|g__Butyrivibrio_2
k__Bacteria|p__Firmicutes|c__Clostridia|o__Clostridiales|f__Lachnospiraceae|g__Catonella
k__Bacteria|p__Firmicutes|c__Clostridia|o__Clostridiales|f__Lachnospiraceae|g__Johnsonella
k__Bacteria|p__Firmicutes|c__Clostridia|o__Clostridiales|f__Lachnospiraceae|g__Lachnoanaerobaculum
k__Bacteria|p__Firmicutes|c__Clostridia|o__Clostridiales|f__Lachnospiraceae|g__Oribacterium
k__Bacteria|p__Firmicutes|c__Clostridia|o__Clostridiales|f__Lachnospiraceae|g__Shuttleworthia
k__Bacteria|p__Firmicutes|c__Clostridia|o__Clostridiales|f__Lachnospiraceae|g__Stomatobaculum
k__Bacteria|p__Firmicutes|c__Clostridia|o__Clostridiales|f__Peptococcaceae|g__Peptococcus
k__Bacteria|p__Firmicutes|c__Clostridia|o__Clostridiales|f__Peptostreptococcaceae|g__Filifactor
k__Bacteria|p__Firmicutes|c__Clostridia|o__Clostridiales|f__Peptostreptococcaceae|g__Peptoclostridium
k__Bacteria|p__Firmicutes|c__Clostridia|o__Clostridiales|f__Peptostreptococcaceae|g__Peptostreptococcus
k__Bacteria|p__Firmicutes|c__Clostridia|o__Clostridiales|f__Ruminococcaceae|g__Ruminococcaceae_UCG-014
k__Bacteria|p__Firmicutes|c__Erysipelotrichia|o__Erysipelotrichales|f__Erysipelotrichaceae|g__Bulleidia
k__Bacteria|p__Firmicutes|c__Erysipelotrichia|o__Erysipelotrichales|f__Erysipelotrichaceae|g__Eggerthia
k__Bacteria|p__Firmicutes|c__Erysipelotrichia|o__Erysipelotrichales|f__Erysipelotrichaceae|g__Erysipelotrichaceae_UCG-006
k__Bacteria|p__Firmicutes|c__Erysipelotrichia|o__Erysipelotrichales|f__Erysipelotrichaceae|g__Solobacterium
k__Bacteria|p__Firmicutes|c__Negativicutes|o__Selenomonadales|f__Veillonellaceae|g__Anaeroglobus
k__Bacteria|p__Firmicutes|c__Negativicutes|o__Selenomonadales|f__Veillonellaceae|g__Dialister
k__Bacteria|p__Firmicutes|c__Negativicutes|o__Selenomonadales|f__Veillonellaceae|g__Megasphaera
k__Bacteria|p__Firmicutes|c__Negativicutes|o__Selenomonadales|f__Veillonellaceae|g__Selenomonas
k__Bacteria|p__Firmicutes|c__Negativicutes|o__Selenomonadales|f__Veillonellaceae|g__Selenomonas_3
k__Bacteria|p__Firmicutes|c__Negativicutes|o__Selenomonadales|f__Veillonellaceae|g__Selenomonas_4
k__Bacteria|p__Firmicutes|c__Negativicutes|o__Selenomonadales|f__Veillonellaceae|g__Veillonella
k__Bacteria|p__Fusobacteria|c__Fusobacteriia|o__Fusobacteriales|f__Fusobacteriaceae|g__Fusobacterium
k__Bacteria|p__Fusobacteria|c__Fusobacteriia|o__Fusobacteriales|f__Leptotrichiaceae|g__Leptotrichia
k__Bacteria|p__Fusobacteria|c__Fusobacteriia|o__Fusobacteriales|f__Leptotrichiaceae|g__Streptobacillus
k__Bacteria|p__Proteobacteria|c__Betaproteobacteria|o__Burkholderiales|f__Burkholderiaceae|g__Lautropia
k__Bacteria|p__Proteobacteria|c__Betaproteobacteria|o__Burkholderiales|f__Burkholderiaceae|g__Ralstonia
k__Bacteria|p__Proteobacteria|c__Betaproteobacteria|o__Burkholderiales|f__Comamonadaceae
k__Bacteria|p__Proteobacteria|c__Betaproteobacteria|o__Burkholderiales|f__Comamonadaceae|g__Ottowia
k__Bacteria|p__Proteobacteria|c__Betaproteobacteria|o__Neisseriales|f__Neisseriaceae|g__Alysiella

k__Bacteria|p__Proteobacteria|c__Betaproteobacteria|o__Neisseriales|f__Neisseriaceae|g__Eikenella
k__Bacteria|p__Proteobacteria|c__Betaproteobacteria|o__Neisseriales|f__Neisseriaceae|g__Kingella
k__Bacteria|p__Proteobacteria|c__Betaproteobacteria|o__Neisseriales|f__Neisseriaceae|g__Neisseria
k__Bacteria|p__Proteobacteria|c__Deltaproteobacteria|o__Desulfobacterales|f__Desulfobulbaceae|g__Desulfobulbus
k__Bacteria|p__Proteobacteria|c__Epsilonproteobacteria|o__Campylobacterales|f__Campylobacteraceae|g__Campylobacter
k__Bacteria|p__Proteobacteria|c__Gammaproteobacteria|o__Cardiobacteriales|f__Cardiobacteriaceae|g__Cardiobacterium
k__Bacteria|p__Proteobacteria|c__Gammaproteobacteria|o__Enterobacteriales|f__Enterobacteriaceae|g__Escherichia/Shigella
k__Bacteria|p__Proteobacteria|c__Gammaproteobacteria|o__Pasteurellales|f__Pasteurellaceae|g__Actinobacillus
k__Bacteria|p__Proteobacteria|c__Gammaproteobacteria|o__Pasteurellales|f__Pasteurellaceae|g__Aggregatibacter
k__Bacteria|p__Proteobacteria|c__Gammaproteobacteria|o__Pasteurellales|f__Pasteurellaceae|g__Haemophilus
k__Bacteria|p__Proteobacteria|c__Gammaproteobacteria|o__Pasteurellales|f__Pasteurellaceae|g__Mannheimia
k__Bacteria|p__Proteobacteria|c__Gammaproteobacteria|o__Pseudomonadales|f__Moraxellaceae|g__Acinetobacter
k__Bacteria|p__Proteobacteria|c__Gammaproteobacteria|o__Pseudomonadales|f__Moraxellaceae|g__Moraxella
k__Bacteria|p__Proteobacteria|c__Gammaproteobacteria|o__Pseudomonadales|f__Pseudomonadaceae|g__Pseudomonas
k__Bacteria|p__Saccharibacteria|c__Unknown_Class|o__Unknown_Order|f__Unknown_Family|g__Candidatus_Saccharimonas
k__Bacteria|p__Spirochaetae|c__Spirochaetes|o__Spirochaetales|f__Spirochaetaceae|g__Treponema_2
k__Bacteria|p__Synergistetes|c__Synergistia|o__Synergistales|f__Synergistaceae|g__Fretibacterium
k__Bacteria|p__Tenericutes|c__Mollicutes|o__Mycoplasmatales|f__Mycoplasmataceae|g__Mycoplasma

Supplementary Table 2: Microbial relative abundance differences at genus level between Case and Control groups

Group	Genus	Mean	Sd	Skewness	Kurtosis	Min	Q1	Mdn	Q3	Max	P	$P_{\text{Benjamini \& Hochberg correct}}$
											0,927	0,936
Case	Abiotrophia	0,00145	0,00314	6,98881	74,21096	0,00000	0,00000	0,00040	0,00171	0,04402		
Control	Abiotrophia	0,00156	0,00403	14,37259	356,31422	0,00000	0,00000	0,00042	0,00171	0,11798		
											0,411	0,511
Case	Acinetobacter	0,00011	0,0005	7,6894	72,3067	0,00000	0,00000	0,00000	0,00000	0,00617		
Control	Acinetobacter	0,00063	0,01368	31,53757	1094,58682	0,00000	0,00000	0,00000	0,00000	0,5204		
											0,022	0,045
Case	Actinobacillus	0,00376	0,0103	7,81802	96,70312	0,00000	0,00000	0,00024	0,00323	0,1692		
Control	Actinobacillus	0,0043	0,01077	6,79786	69,23568	0,00000	0,00000	0,00052	0,00395	0,15799		
											0,392	0,499
Case	Actinomyces	0,0275	0,02083	1,58162	3,94362	0,00000	0,01256	0,02131	0,03758	0,16709		
Control	Actinomyces	0,02833	0,02256	2,26797	8,39238	0,00000	0,01330	0,02243	0,03586	0,19967	0,044	0,078
Case	Aggregatibacter	0,00616	0,01124	4,00209	21,61852	0,00000	0,00000	0,00203	0,00796	0,09765		
Control	Aggregatibacter	0,00499	0,00949	5,56108	54,1193	0,00000	0,00000	0,00149	0,00593	0,14504		
											0,009	0,02
Case	Alloprevotella	0,0207	0,0201	1,74022	3,61189	0,00000	0,00649	0,01456	0,02827	0,11862		
Control	Alloprevotella	0,01936	0,02149	3,04844	19,39603	0,00000	0,00534	0,01275	0,02567	0,27771		
											0,58	0,66
Case	Alloscardovia	0,00052	0,00198	10,63581	157,8468	0,00000	0,00000	0,00000	0,00026	0,03591		
Control	Alloscardovia	0,00048	0,00196	11,00627	155,04275	0,00000	0,00000	0,00000	0,00026	0,03606		
											<0.001	<0.001
Case	Alysiella	0,00007	0,00038	12,15352	197,24006	0,00000	0,00000	0,00000	0,00000	0,00736		
Control	Alysiella	0,00008	0,00031	9,76671	142,22784	0,00000	0,00000	0,00000	0,00000	0,00654		
											<0.001	<0.001
Case	Anaeroglobus	0,00139	0,00301	5,265	40,05599	0,00000	0,00000	0,00038	0,00147	0,03715		
Control	Anaeroglobus	0,00096	0,00288	9,68622	134,46708	0,00000	0,00000	0,00017	0,00079	0,05509		
											0,531	0,624
Case	Atopobium	0,00773	0,00863	2,38872	7,68046	0,00000	0,00217	0,00472	0,01019	0,06751		
Control	Atopobium	0,00718	0,00827	4,2537	38,70705	0,00000	0,00225	0,00481	0,00926	0,12784		
											0,063	0,111
Case	Bacteroides	0,0007	0,00346	9,49903	102,41148	0,00000	0,00000	0,00000	0,00028	0,04971		
Control	Bacteroides	0,00047	0,00394	35,01793	1401,98047	0,00000	0,00000	0,00000	0,00017	0,16272		
											0,035	0,067
Case	Bergeyella	0,00254	0,00572	8,74104	101,62362	0,00000	0,00056	0,00131	0,00254	0,0838		
Control	Bergeyella	0,00301	0,00772	9,07761	107,46262	0,00000	0,00063	0,00146	0,00275	0,12367		
											0,002	0,006
Case	Bifidobacterium	0,00107	0,00229	4,14209	23,89956	0,00000	0,00000	0,00017	0,00097	0,02454		
Control	Bifidobacterium	0,001	0,00383	13,57851	251,75659	0,00000	0,00000	0,00010	0,00069	0,09345		
											<0.001	<0.001
Case	Bulleidia	0,00057	0,00144	6,84261	68,65699	0,00000	0,00000	0,00005	0,00056	0,02035		
Control	Bulleidia	0,00046	0,00118	5,31858	38,98056	0,00000	0,00000	0,00000	0,00037	0,01446		
											0,719	0,777
Case	Butyrivibrio_2	0,0002	0,00063	9,00478	113,67844	0,00000	0,00000	0,00000	0,00017	0,01038		
Control	Butyrivibrio_2	0,00019	0,00049	5,48981	39,90398	0,00000	0,00000	0,00000	0,00019	0,00537		
											0,003	0,008
Case	Campylobacter	0,00548	0,00511	2,67037	12,55342	0,00000	0,00228	0,00399	0,00695	0,05165		
Control	Campylobacter	0,00484	0,00479	4,50745	45,67424	0,00000	0,00199	0,00377	0,00626	0,08221		
											0,389	0,499
Case	Candidatus_Saccharimonas	0,00004	0,00011	7,31398	97,65245	0,00000	0,00000	0,00000	0,00004	0,00182		
Control	Candidatus_Saccharimonas	0,00004	0,0001	4,39718	27,18513	0,00000	0,00000	0,00000	0,00005	0,00106		
											0,115	0,184
Case	Capnocytophaga	0,0033	0,00458	5,18921	46,99353	0,00000	0,00075	0,00200	0,00415	0,06218		
Control	Capnocytophaga	0,00292	0,00383	4,71047	42,64465	0,00000	0,00073	0,00180	0,00365	0,06076		
											0,218	0,329
Case	Cardiobacterium	0,00045	0,0009	6,57315	72,46874	0,00000	0,00000	0,00018	0,00054	0,01382		
Control	Cardiobacterium	0,00047	0,00087	5,55751	52,21902	0,00000	0,00000	0,00019	0,00054	0,01392		
											0,003	0,008
Case	Catonella	0,00089	0,00127	3,61063	20,08449	0,00000	0,00018	0,00049	0,00107	0,01283		
Control	Catonella	0,00073	0,00098	3,21847	16,28165	0,00000	0,00011	0,00043	0,00096	0,01016		
											0,427	0,525
Case	Cloacibacterium	0,00024	0,00222	17,00413	311,34045	0,00000	0,00000	0,00000	0,00000	0,04428		
Control	Cloacibacterium	0,00054	0,00927	31,11149	1041,0322	0,00000	0,00000	0,00000	0,00000	0,34103		
											0,383	0,499
Case	Corynebacterium	0,00476	0,00627	4,02846	23,95423	0,00000	0,00133	0,00289	0,00589	0,05844		
Control	Corynebacterium	0,00454	0,00645	7,61279	125,17644	0,00000	0,00124	0,00277	0,00559	0,14661		
											0,096	0,16
Case	Cryptobacterium	0,00014	0,00034	4,53381	26,42072	0,00000	0,00000	0,00000	0,00012	0,00336		
Control	Cryptobacterium	0,00013	0,00045	11,07417	171,1547	0,00000	0,00000	0,00000	0,00008	0,00917		
											<0.001	<0.001
Case	Defluviitaleaceae_UCG-011	0,00041	0,001	4,71656	30,54379	0,00000	0,00000	0,00000	0,00034	0,01054		
Control	Defluviitaleaceae_UCG-011	0,00024	0,00075	7,28971	73,18238	0,00000	0,00000	0,00000	0,00016	0,01148		
											<0.001	<0.001
Case	Desulfobulbus	0,0002	0,00059	5,78925	39,49057	0,00000	0,00000	0,00000	0,00014	0,00594		
Control	Desulfobulbus	0,00013	0,00055	16,8527	444,39931	0,00000	0,00000	0,00000	0,00003	0,01676		
											<0.001	<0.001
Case	Dialister	0,00306	0,00544	9,19275	140,71831	0,00000	0,00052	0,00156	0,00374	0,1004		
Control	Dialister	0,00232	0,00373	5,03658	41,72066	0,00000	0,00032	0,00112	0,00285	0,04996		
											<0.001	<0.001
Case	Eggerthia	0,00019	0,00057	6,50931	57,3311	0,00000	0,00000	0,00000	0,00011	0,00747		
Control	Eggerthia	0,00012	0,00049	9,74341	135,52781	0,00000	0,00000	0,00000	0,00004	0,01008		
											0,008	0,018

Case	Eikenella	0,00021	0,00085	17,56631	386,2731	0,00000	0,00000	0,00000	0,00022	0,01999		
Control	Eikenella	0,00028	0,00451	36,55588	1447,00611	0,00000	0,00000	0,00000	0,00015	0,18673		
											0,006	0,014
Case	Erysipelotrichaceae_UCG-006	0,00008	0,00021	6,18331	51,83577	0,00000	0,00000	0,00000	0,00007	0,00256		
Control	Erysipelotrichaceae_UCG-006	0,00007	0,0002	6,27523	55,44782	0,00000	0,00000	0,00000	0,00005	0,00261		
											0,384	0,499
Case	Escherichia/Shigella	0,00029	0,00383	24,00714	614,56054	0,00000	0,00000	0,00000	0,00000	0,10046		
Control	Escherichia/Shigella	0,00032	0,0043	24,32926	703,32104	0,00000	0,00000	0,00000	0,00000	0,14492		
											<0.001	<0.001
Case	Family_XIII_UCG-001	0,00016	0,0003	3,76417	18,91733	0,00000	0,00000	0,00003	0,00018	0,00279		
Control	Family_XIII_UCG-001	0,0001	0,00022	5,78981	53,99604	0,00000	0,00000	0,00000	0,00011	0,00345		
											<0.001	<0.001
Case	Filifactor	0,00262	0,00473	3,10391	12,07879	0,00000	0,00000	0,00062	0,00295	0,03498		
Control	Filifactor	0,00148	0,00321	4,41851	27,77403	0,00000	0,00000	0,00012	0,00152	0,03664		
											<0.001	<0.001
Case	Fretibacterium	0,00099	0,00201	6,15284	62,50018	0,00000	0,00000	0,00029	0,00110	0,02886		
Control	Fretibacterium	0,00062	0,00147	6,08467	55,06034	0,00000	0,00000	0,00011	0,00061	0,01986		
											<0.001	<0.001
Case	Fusobacterium	0,03208	0,02592	1,62755	3,9075	0,00000	0,01362	0,02588	0,04448	0,19246		
Control	Fusobacterium	0,02654	0,02293	2,30494	11,45978	0,00000	0,01045	0,02151	0,03593	0,26484		
											0,102	0,167
Case	Gemella	0,04121	0,03566	1,67986	4,45614	0,00000	0,01490	0,03270	0,05876	0,26604		
Control	Gemella	0,04274	0,03517	1,84951	6,91211	0,00000	0,01799	0,03554	0,05912	0,36225		
											0,012	0,026
Case	Granulicatella	0,02205	0,01471	1,41001	3,50957	0,00000	0,01173	0,02027	0,02925	0,09597		
Control	Granulicatella	0,02389	0,01638	2,15287	13,90038	0,00000	0,01285	0,02106	0,03129	0,22611		
											<0.001	<0.001
Case	Haemophilus	0,04691	0,04498	2,56043	12,12206	0,00000	0,01475	0,03788	0,06497	0,4094		
Control	Haemophilus	0,05364	0,04487	1,40406	3,7006	0,00000	0,01896	0,04548	0,07643	0,41312		
											<0.001	<0.001
Case	Incertae_Sedis	0,00099	0,00226	4,0894	22,9056	0,00000	0,00000	0,00000	0,00087	0,02314		
Control	Incertae_Sedis	0,00056	0,00169	5,68493	44,68854	0,00000	0,00000	0,00000	0,00025	0,02524		
											0,004	0,01
Case	Johnsonella	0,00029	0,00125	16,08803	319,46952	0,00000	0,00000	0,00000	0,00024	0,02753		
Control	Johnsonella	0,00023	0,00064	5,62117	43,45952	0,00000	0,00000	0,00000	0,00016	0,00791		
											0,274	0,391
Case	Kingella	0,00055	0,00102	4,43596	27,19733	0,00000	0,00000	0,00021	0,00065	0,00995		
Control	Kingella	0,00065	0,00307	37,17691	1564,45686	0,00000	0,00000	0,00023	0,00063	0,13061		
											0,556	0,64
Case	Lachnoanaerobaculum	0,00236	0,00256	3,02307	16,54662	0,00000	0,00070	0,00175	0,00305	0,02662		
Control	Lachnoanaerobaculum	0,00246	0,00281	3,82777	29,23402	0,00000	0,00071	0,00175	0,00322	0,03666		
											0,036	0,068
Case	Lactobacillus	0,00675	0,02352	6,03994	41,93818	0,00000	0,00000	0,00016	0,00292	0,22307		
Control	Lactobacillus	0,00614	0,0265	9,10227	107,72838	0,00000	0,00000	0,00011	0,00161	0,48538		
											0,24	0,355
Case	Lautropia	0,00107	0,00264	7,6073	80,05195	0,00000	0,00000	0,00027	0,00111	0,03696		
Control	Lautropia	0,00106	0,00218	5,25058	41,69322	0,00000	0,00000	0,00032	0,00118	0,03164		
											0,858	0,883
Case	Leptotrichia	0,01984	0,02186	5,4126	63,51367	0,00000	0,00692	0,01410	0,02562	0,34241		
Control	Leptotrichia	0,02013	0,02116	2,85037	13,75322	0,00000	0,00670	0,01394	0,02636	0,22771		
											0,242	0,355
Case	Mannheimia	0,00104	0,00507	10,93548	156,58606	0,00000	0,00000	0,00000	0,00000	0,09035		
Control	Mannheimia	0,00102	0,00414	10,01586	165,05501	0,00000	0,00000	0,00000	0,00000	0,096		
											0,706	0,771
Case	Megasphaera	0,00605	0,00746	2,53193	9,7055	0,00000	0,00115	0,00338	0,00831	0,06581		
Control	Megasphaera	0,00593	0,00689	2,19523	7,00592	0,00000	0,00113	0,00355	0,00821	0,05727		
											0,007	0,016
Case	Mogibacterium	0,00319	0,00325	1,93601	5,5055	0,00000	0,00087	0,00211	0,00458	0,02282		
Control	Mogibacterium	0,00275	0,00281	2,17301	8,8852	0,00000	0,00075	0,00192	0,00394	0,02949		
											0,819	0,851
Case	Moraxella	0,00017	0,00108	10,36841	126,86377	0,00000	0,00000	0,00000	0,00000	0,0168		
Control	Moraxella	0,00027	0,00392	25,6013	736,17967	0,00000	0,00000	0,00000	0,00000	0,13143		
											<0.001	<0.001
Case	Mycoplasma	0,00104	0,00222	5,29412	41,99663	0,00000	0,00000	0,00026	0,00107	0,02773		
Control	Mycoplasma	0,00066	0,0015	5,58435	49,42985	0,00000	0,00000	0,00011	0,00062	0,02192		
											0,028	0,055
Case	Neisseria	0,02217	0,03211	2,54246	9,16146	0,00000	0,00138	0,00888	0,03087	0,26879		
Control	Neisseria	0,02436	0,03356	2,44029	8,12448	0,00000	0,00199	0,01125	0,03347	0,29119		
											<0.001	<0.001
Case	Olsenella	0,00021	0,00048	5,06556	39,57276	0,00000	0,00000	0,00000	0,00022	0,006		
Control	Olsenella	0,00015	0,0006	11,10529	167,68798	0,00000	0,00000	0,00000	0,00009	0,01251		
											0,404	0,509
Case	Oribacterium	0,00314	0,00317	2,46862	9,87922	0,00000	0,00113	0,00228	0,00419	0,02587		
Control	Oribacterium	0,00325	0,00323	2,34675	9,58559	0,00000	0,00100	0,00249	0,00453	0,02845		
											0,379	0,499
Case	Ottowia	0,00028	0,00434	26,36998	711,73746	0,00000	0,00000	0,00000	0,00000	0,118		
Control	Ottowia	0,00031	0,00493	37,86992	1567,43896	0,00000	0,00000	0,00000	0,00000	0,20902		
											<0.001	<0.001
Case	Parascardovia	0,00036	0,00147	10,61431	155,91273	0,00000	0,00000	0,00000	0,00015	0,02684		
Control	Parascardovia	0,00025	0,00116	12,47493	245,75322	0,00000	0,00000	0,00000	0,00000	0,03022		
											<0.001	<0.001
Case	Parvimonas	0,00728	0,00898	2,73287	12,25746	0,00000	0,00138	0,00406	0,01002	0,08419		
Control	Parvimonas	0,00535	0,00747	3,72706	23,48596	0,00000	0,00087	0,00285	0,00705	0,09427		

											<0.001	<0.001
Case	Peptoclostridium	0,0004	0,00092	6,04997	55,37572	0,00000	0,00000	0,00007	0,00040	0,01257		
Control	Peptoclostridium	0,00024	0,0005	4,17801	23,96588	0,00000	0,00000	0,00000	0,00026	0,00506		
											<0.001	<0.001
Case	Peptococcus	0,00092	0,00167	5,5009	59,40977	0,00000	0,00000	0,00026	0,00111	0,02481		
Control	Peptococcus	0,0006	0,00122	6,66429	83,46825	0,00000	0,00000	0,00015	0,00074	0,02381		
											<0.001	<0.001
Case	Peptostreptococcus	0,00436	0,00641	3,59405	18,9345	0,00000	0,00045	0,00231	0,00548	0,05677		
Control	Peptostreptococcus	0,00319	0,0045	2,69247	10,42145	0,00000	0,00022	0,00147	0,00437	0,04309		
											<0.001	<0.001
Case	Phocaeicola	0,00017	0,00048	6,89863	66,32745	0,00000	0,00000	0,00000	0,00014	0,00616		
Control	Phocaeicola	0,00011	0,0004	10,5768	159,46636	0,00000	0,00000	0,00000	0,00007	0,00836		
											<0.001	<0.001
Case	Porphyromonas	0,02031	0,02174	3,1809	22,88843	0,00000	0,00486	0,01436	0,02938	0,26373		
Control	Porphyromonas	0,01616	0,01765	2,31545	8,65247	0,00000	0,00373	0,01087	0,02292	0,16106		
											0,03	0,058
Case	Prevotella	0,02611	0,0229	2,67684	15,02678	0,00000	0,01049	0,02058	0,03534	0,24231		
Control	Prevotella	0,02385	0,02022	2,00581	7,64631	0,00000	0,00971	0,01949	0,03288	0,1982		
											<0.001	<0.001
Case	Prevotella_2	0,0017	0,00218	4,22623	35,88457	0,00000	0,00035	0,00103	0,00227	0,02873		
Control	Prevotella_2	0,00147	0,0022	6,59146	86,46095	0,00000	0,00024	0,00085	0,00193	0,04093		
											0,084	0,143
Case	Prevotella_6	0,0065	0,01051	8,0016	113,0529	0,00000	0,00119	0,00366	0,00793	0,18411		
Control	Prevotella_6	0,00666	0,00823	3,07695	16,02295	0,00000	0,00145	0,00396	0,00911	0,08324		
											0,586	0,66
Case	Prevotella_7	0,08186	0,05721	1,03384	1,05004	0,00000	0,03967	0,07178	0,11148	0,3267		
Control	Prevotella_7	0,08248	0,05645	1,02684	1,39559	0,00000	0,04039	0,07247	0,11492	0,37674		
											0,73	0,778
Case	Pseudomonas	0,00042	0,00554	22,39227	541,40677	0,00000	0,00000	0,00000	0,00000	0,14009		
Control	Pseudomonas	0,00028	0,00653	42,73664	1883,28994	0,00000	0,00000	0,00000	0,00000	0,29009		
											<0.001	<0.001
Case	Pseudoramibacter	0,00014	0,00033	4,05595	20,37649	0,00000	0,00000	0,00000	0,00011	0,00304		
Control	Pseudoramibacter	0,00011	0,00053	13,26598	245,47493	0,00000	0,00000	0,00000	0,00003	0,01246		
											0,127	0,198
Case	Ralstonia	0,00009	0,00093	23,95509	614,64976	0,00000	0,00000	0,00000	0,00000	0,02443		
Control	Ralstonia	0,00021	0,00456	36,10253	1382,04744	0,00000	0,00000	0,00000	0,00000	0,18522		
											<0.001	<0.001
Case	Rothia	0,10923	0,08582	2,0561	7,51688	0,00000	0,05132	0,09126	0,14205	0,67375		
Control	Rothia	0,12537	0,09459	2,28593	8,93384	0,00000	0,06443	0,10335	0,16214	0,88493		
											0,291	0,404
Case	Ruminococcaceae_UCG-014	0,00151	0,00205	3,37068	17,76243	0,00000	0,00017	0,00089	0,00206	0,0188		
Control	Ruminococcaceae_UCG-014	0,00162	0,00235	4,8006	39,21707	0,00000	0,00023	0,00093	0,00217	0,03063		
											0,041	0,074
Case	Scardovia	0,00181	0,00595	7,72775	78,03966	0,00000	0,00000	0,00022	0,00107	0,08337		
Control	Scardovia	0,00153	0,00689	17,89196	471,56868	0,00000	0,00000	0,00015	0,00089	0,21425		
											<0.001	<0.001
Case	Selenomonas	0,0011	0,00218	4,59647	28,36367	0,00000	0,00006	0,00038	0,00117	0,02026		
Control	Selenomonas	0,00069	0,00165	8,86785	126,1667	0,00000	0,00000	0,00021	0,00066	0,03236		
											0,023	0,046
Case	Selenomonas_3	0,00172	0,00259	4,72771	33,76522	0,00000	0,00033	0,00088	0,00216	0,02873		
Control	Selenomonas_3	0,00154	0,00241	6,29292	77,82857	0,00000	0,00025	0,00083	0,00188	0,04447		
											<0.001	<0.001
Case	Selenomonas_4	0,00017	0,00041	5,29092	38,36786	0,00000	0,00000	0,00000	0,00017	0,00452		
Control	Selenomonas_4	0,0001	0,0003	9,68253	156,92717	0,00000	0,00000	0,00000	0,00008	0,00693		
											0,003	0,008
Case	Shuttleworthia	0,00026	0,00055	4,08513	21,70675	0,00000	0,00000	0,00000	0,00029	0,00488		
Control	Shuttleworthia	0,00021	0,00061	9,37015	143,25972	0,00000	0,00000	0,00000	0,00019	0,01316		
											0,128	0,198
Case	Solobacterium	0,0032	0,00359	2,85734	14,28117	0,00000	0,00088	0,00203	0,00434	0,03454		
Control	Solobacterium	0,00298	0,00347	3,23883	17,11573	0,00000	0,00083	0,00199	0,00388	0,03463		
											0,375	0,499
Case	Staphylococcus	0,00025	0,00458	27,09819	739,52289	0,00000	0,00000	0,00000	0,00000	0,12586		
Control	Staphylococcus	0,00008	0,00043	13,24773	240,83775	0,00000	0,00000	0,00000	0,00000	0,0103		
											0,624	0,696
Case	Stomatobaculum	0,00282	0,00282	1,87194	4,61041	0,00000	0,00079	0,00202	0,00379	0,0193		
Control	Stomatobaculum	0,00289	0,00302	2,56962	11,57809	0,00000	0,00086	0,00209	0,00402	0,02915		
											0,04	0,074
Case	Streptobacillus	0,00056	0,00313	9,46587	99,22419	0,00000	0,00000	0,00000	0,00000	0,04155		
Control	Streptobacillus	0,00032	0,00171	9,82092	120,53949	0,00000	0,00000	0,00000	0,00000	0,02977		
											0,103	0,167
Case	Streptococcus	0,31696	0,1297	0,82301	2,96658	0,00000	0,24283	0,30361	0,38473	0,92385		
Control	Streptococcus	0,32174	0,12683	0,43715	2,13934	0,00000	0,24855	0,31890	0,38961	0,95546		
											<0.001	<0.001
Case	Tannerella	0,00152	0,00265	8,03257	109,35685	0,00000	0,00022	0,00077	0,00186	0,04542		
Control	Tannerella	0,00105	0,00168	4,08304	24,91582	0,00000	0,00009	0,00048	0,00133	0,01964		
											<0.001	<0.001
Case	Treponema_2	0,00681	0,01033	3,19964	16,98753	0,00000	0,00052	0,00270	0,00872	0,10927		
Control	Treponema_2	0,0045	0,00893	5,52746	52,66113	0,00000	0,00014	0,00107	0,00504	0,12706		
											0,247	0,357
Case	Veillonella	0,06095	0,03686	1,3639	4,09779	0,00000	0,03358	0,05605	0,08131	0,25812		
Control	Veillonella	0,06235	0,03594	1,00246	1,83101	0,00000	0,03682	0,05744	0,08050	0,27892		

Supplementary Table 3: Linear discriminant analysis (LDA) of microbial communities between Case and Control groups

Biomarkernames	Log10AverageAbundance	EnrichedGroups	LDA	KW_Pvalue
k__Bacteria.p__Actinobacteria.c__Actinobacteria.o__Micrococcales.f__Micrococcaceae.g__Rothia	5,099	Control	3,926	0,000E+00
k__Bacteria.p__Proteobacteria.c__Gammaproteobacteria.o__Pasteurellales.f__Pasteurellaceae.g__Haemophi	4,731	Control	3,549	5,000E-05
k__Bacteria.p__Fusobacteria.c__Fusobacteriia.o__Fusobacteriales.f__Fusobacteriaceae.g__Fusobacterium	4,509	Case	3,476	0,000E+00
k__Bacteria.p__Bacteroidetes.c__Bacteroidia.o__Bacteroidales.f__Porphyromonadaceae.g__Porphyromonas	4,310	Case	3,338	0,000E+00
k__Bacteria.p__Bacteroidetes.c__Bacteroidia.o__Bacteroidales.f__Prevotellaceae.g__Prevotella	4,420	Case	3,130	2,694E-02
k__Bacteria.p__Firmicutes.c__Clostridia.o__Clostridiales.f__Peptostreptococcaceae	3,871	Case	3,104	0,000E+00
k__Bacteria.p__Spirochaetae.c__Spirochaetes.o__Spirochaetales.f__Spirochaetaceae.g__Treponema_2	3,837	Case	3,066	0,000E+00
k__Bacteria.p__Spirochaetae	3,837	Case	3,066	0,000E+00
k__Bacteria.p__Spirochaetae.c__Spirochaetes	3,837	Case	3,066	0,000E+00
k__Bacteria.p__Spirochaetae.c__Spirochaetes.o__Spirochaetales	3,837	Case	3,066	0,000E+00
k__Bacteria.p__Spirochaetae.c__Spirochaetes.o__Spirochaetales.f__Spirochaetaceae	3,837	Case	3,066	0,000E+00
k__Bacteria.p__Proteobacteria.c__Betaproteobacteria.o__Neisseriales	4,407	Control	3,056	4,742E-02
k__Bacteria.p__Proteobacteria.c__Betaproteobacteria.o__Neisseriales.f__Neisseriaceae.g__Neisseria	4,388	Control	3,035	2,939E-02
k__Bacteria.p__Firmicutes.c__Clostridia.o__Clostridiales.f__Family_XI.g__Parvimonas	3,864	Case	3,027	0,000E+00
k__Bacteria.p__Firmicutes.c__Clostridia.o__Clostridiales.f__Family_XI	3,864	Case	3,027	0,000E+00
k__Bacteria.p__Firmicutes.c__Bacilli.o__Lactobacillales.f__Carnobacteriaceae.g__Granulicatella	4,380	Control	2,968	1,234E-02
k__Bacteria.p__Firmicutes.c__Bacilli.o__Lactobacillales.f__Carnobacteriaceae	4,380	Control	2,968	1,234E-02
k__Bacteria.p__Bacteroidetes.c__Bacteroidia.o__Bacteroidales.f__Prevotellaceae.g__Alloprevotella	4,318	Case	2,878	8,784E-03
k__Bacteria.p__Fusobacteria.c__Fusobacteriia.o__Fusobacteriales.f__Leptotrichiaceae	3,614	Case	2,867	3,631E-03
k__Bacteria.p__Proteobacteria.c__Gammaproteobacteria.o__Pasteurellales.f__Pasteurellaceae.g__Aggregatil	3,792	Case	2,794	4,231E-02
k__Bacteria.p__Firmicutes.c__Clostridia.o__Clostridiales.f__Peptostreptococcaceae.g__Peptostreptococcus	3,642	Case	2,785	0,000E+00
k__Bacteria.p__Firmicutes.c__Bacilli.o__Lactobacillales.f__Lactobacillaceae.g__Lactobacillus	3,837	Case	2,779	3,759E-02
k__Bacteria.p__Firmicutes.c__Bacilli.o__Lactobacillales.f__Lactobacillaceae	3,837	Case	2,779	3,759E-02
k__Bacteria.p__Firmicutes.c__Clostridia.o__Clostridiales.f__Peptostreptococcaceae.g__Filifactor	3,422	Case	2,764	0,000E+00
k__Bacteria.p__Bacteroidetes.c__Bacteroidia.o__Bacteroidales.f__Prevotellaceae	3,325	Case	2,666	0,000E+00
k__Bacteria.p__Firmicutes.c__Negativicutes.o__Selenomonadales.f__Veillonellaceae.g__Dialister	3,488	Case	2,624	0,000E+00
k__Bacteria.p__Proteobacteria.c__Gammaproteobacteria.o__Pasteurellales.f__Pasteurellaceae.g__Actinobaci	3,634	Control	2,549	2,206E-02
k__Bacteria.p__Proteobacteria.c__Epsilonproteobacteria.o__Campylobacterales.f__Campylobacteraceae.g__(	3,741	Case	2,532	3,545E-03
k__Bacteria.p__Proteobacteria.c__Epsilonproteobacteria	3,741	Case	2,532	3,545E-03
k__Bacteria.p__Proteobacteria.c__Epsilonproteobacteria.o__Campylobacterales	3,741	Case	2,532	3,545E-03
k__Bacteria.p__Proteobacteria.c__Epsilonproteobacteria.o__Campylobacterales.f__Campylobacteraceae	3,741	Case	2,532	3,545E-03
k__Bacteria.p__Firmicutes.c__Clostridia.o__Clostridiales.f__Family_XIII.g___Eubacterium__nodatum_group	3,382	Case	2,496	3,000E-05
k__Bacteria.p__Actinobacteria.c__Actinobacteria.o__Bifidobacteriales	3,577	Case	2,437	9,367E-03
k__Bacteria.p__Actinobacteria.c__Actinobacteria.o__Bifidobacteriales.f__Bifidobacteriaceae	3,577	Case	2,437	9,367E-03
k__Bacteria.p__Bacteroidetes.c__Flavobacteriia.o__Flavobacteriales.f__Flavobacteriaceae.g__Bergeyella	3,480	Control	2,401	3,579E-02
k__Bacteria.p__Bacteroidetes.c__Bacteroidia.o__Bacteroidales.f__Porphyromonadaceae.g__Tannerella	3,185	Case	2,399	0,000E+00
k__Bacteria.p__Firmicutes.c__Erysipelotrichia	3,607	Case	2,370	1,962E-02
k__Bacteria.p__Firmicutes.c__Erysipelotrichia.o__Erysipelotrichales	3,607	Case	2,370	1,962E-02
k__Bacteria.p__Firmicutes.c__Erysipelotrichia.o__Erysipelotrichales.f__Erysipelotrichaceae	3,607	Case	2,370	1,962E-02
k__Bacteria.p__Firmicutes.c__Clostridia.o__Clostridiales.f__Family_XIII.g___Eubacterium__brachy_group	3,227	Case	2,367	2,227E-04
k__Bacteria.p__Firmicutes.c__Clostridia.o__Clostridiales.f__Family_XIII.g__Incertae_Sedis	2,999	Case	2,362	0,000E+00
k__Bacteria.p__Tenericutes	3,097	Case	2,359	0,000E+00
k__Bacteria.p__Tenericutes.c__Mollicutes	3,097	Case	2,359	0,000E+00
k__Bacteria.p__Firmicutes.c__Clostridia.o__Clostridiales.f__Family_XIII.g__Mogibacterium	3,506	Case	2,358	6,472E-03
k__Bacteria.p__Firmicutes.c__Negativicutes.o__Selenomonadales.f__Veillonellaceae.g__Selenomonas	3,045	Case	2,322	0,000E+00
k__Bacteria.p__Tenericutes.c__Mollicutes.o__Mycoplasmatales.f__Mycoplasmataceae.g__Mycoplasma	3,021	Case	2,316	0,000E+00
k__Bacteria.p__Tenericutes.c__Mollicutes.o__Mycoplasmatales	3,021	Case	2,316	0,000E+00
k__Bacteria.p__Tenericutes.c__Mollicutes.o__Mycoplasmatales.f__Mycoplasmataceae	3,021	Case	2,316	0,000E+00
k__Bacteria.p__Firmicutes.c__Negativicutes.o__Selenomonadales.f__Veillonellaceae.g__Anaeroglobus	3,145	Case	2,311	0,000E+00
k__Bacteria.p__Synergistetes.c__Synergistia.o__Synergistales.f__Synergistaceae.g__Fretibacterium	2,998	Case	2,292	0,000E+00
k__Bacteria.p__Synergistetes	2,998	Case	2,292	0,000E+00
k__Bacteria.p__Synergistetes.c__Synergistia	2,998	Case	2,292	0,000E+00
k__Bacteria.p__Synergistetes.c__Synergistia.o__Synergistales	2,998	Case	2,292	0,000E+00
k__Bacteria.p__Synergistetes.c__Synergistia.o__Synergistales.f__Synergistaceae	2,998	Case	2,292	0,000E+00
k__Bacteria.p__Firmicutes.c__Clostridia.o__Clostridiales.f__Peptococcaceae.g__Peptococcus	2,964	Case	2,230	1,000E-05
k__Bacteria.p__Firmicutes.c__Clostridia.o__Clostridiales.f__Peptococcaceae	2,964	Case	2,230	1,000E-05
k__Bacteria.p__Actinobacteria.c__Actinobacteria.o__Bifidobacteriales.f__Bifidobacteriaceae.g__Scardovia	3,259	Case	2,228	4,103E-02
k__Bacteria.p__Bacteroidetes.c__Bacteroidia.o__Bacteroidales.f__Porphyromonadaceae	3,176	Case	2,144	2,702E-03
k__Bacteria.p__Bacteroidetes.c__Bacteroidia.o__Bacteroidales.f__Prevotellaceae.g__Prevotella_2	3,232	Case	2,128	2,314E-04
k__Bacteria.p__Fusobacteria.c__Fusobacteriia.o__Fusobacteriales.f__Leptotrichiaceae.g__Streptobacillus	2,747	Case	2,010	4,010E-02

Supplementary Table 4: The difference genus between the Wilcoxon test,LEfSe, and STMAP method.

Wilcoxon	LDA	STAMP	Common elements
Actinobacillus	Actinobacillus	Aggregatibacter	Anaeroglobus
Alloprevotella	Aggregatibacter	Anaeroglobus	Campylobacter
Alysiella	Alloprevotella	Campylobacter	Dialister
Anaeroglobus	Anaeroglobus	Catonella	Filifactor
Bifidobacterium	Bergeyella	Defluviitaleaceae_UCG-011	Fretibacterium
Bulleidia	Campylobacter	Desulfobulbus	Fusobacterium
Campylobacter	Dialister	Dialister	Granulicatella
Catonella	Filifactor	Eggerthia	Haemophilus
Defluviitaleaceae_UCG-011	Fretibacterium	Family_XIII_UCG-001	Mogibacterium
Desulfobulbus	Fusobacterium	Filifactor	Mycoplasma
Dialister	Granulicatella	Fretibacterium	Parvimonas
Eggerthia	Haemophilus	Fusobacterium	Peptococcus
Eikenella	Lactobacillus	Granulicatella	Peptostreptococcus
Erysipelotrichaceae_UCG-006	Mogibacterium	Haemophilus	Porphyromonas
Family_XIII_UCG-001	Mycoplasma	Mogibacterium	Prevotella_2
Filifactor	Neisseria	Mycoplasma	Rothia
Fretibacterium	Parvimonas	Olsenella	Selenomonas
Fusobacterium	Peptococcus	Parvimonas	Tannerella
Granulicatella	Peptostreptococcus	Peptoclostridium	Treponema_2
Haemophilus	Porphyromonas	Peptococcus	
Johnsonella	Prevotella	Peptostreptococcus	
Mogibacterium	Prevotella_2	Phocaeicola	
Mycoplasma	Rothia	Porphyromonas	
Olsenella	Scardovia	Prevotella	
Parascardovia	Selenomonas	Prevotella_2	
Parvimonas	Streptobacillus	Rothia	
Peptoclostridium	Tannerella	Selenomonas	
Peptococcus	Treponema_2	Selenomonas_4	
Peptostreptococcus		Tannerella	
Phocaeicola		Treponema_2	
Porphyromonas			
Prevotella_2			
Pseudoramibacter			
Rothia			
Selenomonas			
Selenomonas_3			
Selenomonas_4			
Shuttleworthia			
Tannerella			
Treponema_2			
