# Supplementary material for: Characterising oral microbial signatures for periodontal disease in the NHANES population
Source: Acta Odontol Scand. 2026 Mar 30;85:45662. doi: 10.2340/aos.v85.45662 (PMC13058696; doi:10.2340/aos.v85.45662)

Supplementary material has been published as submitted. It has not been copyedited or typeset by Acta Odontologica Scandinavica.

Supplementary Figures

Supplementary Figure 1: Rarefaction curve of (A) Faith's Phylogenetic Diversity index, (B) Inverse-Simpson Index and (C) Shannon-Weiner index.

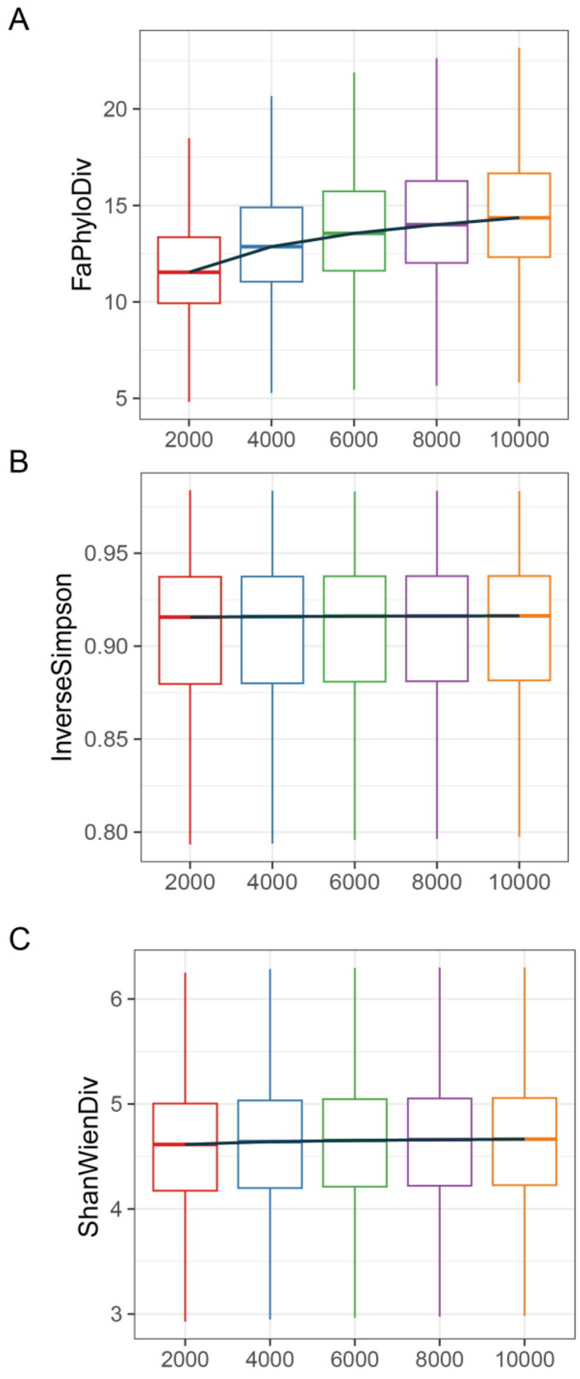

Supplementary Figure 2: alpha diversity of (A) Faith's Phylogenetic Diversity index, (B) Inverse-Simpson Index and (C) Shannon-Weiner index.

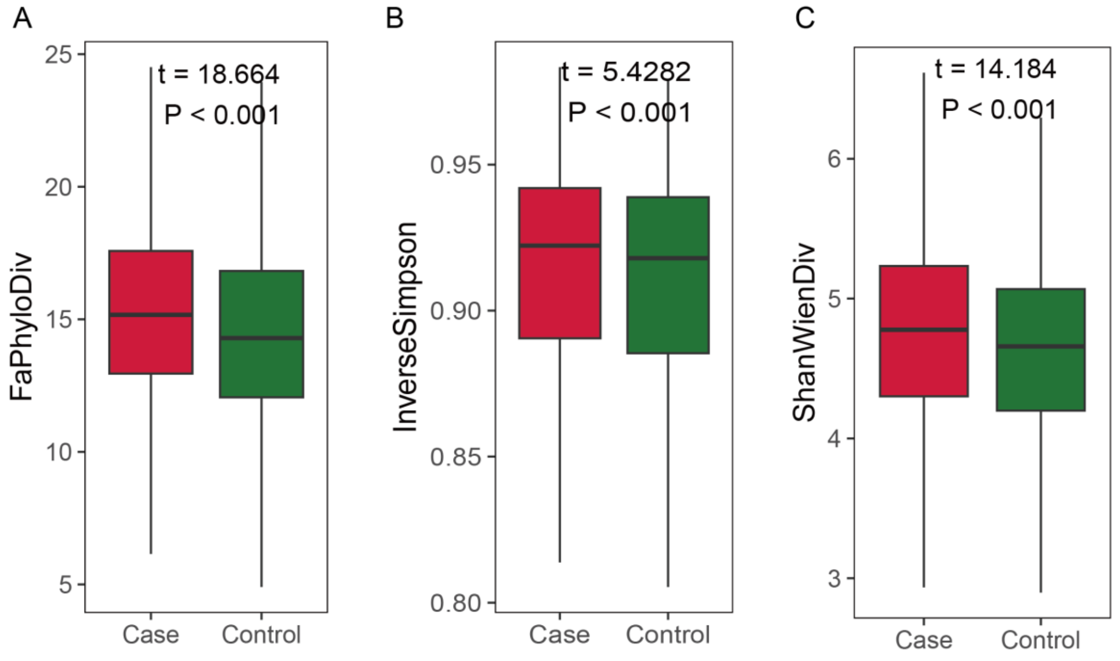

Supplementary Figure 3: Principal components analysis of (A) bray-curtis distances and (B) unweighted weighted UNIFRAC distance across all samples.

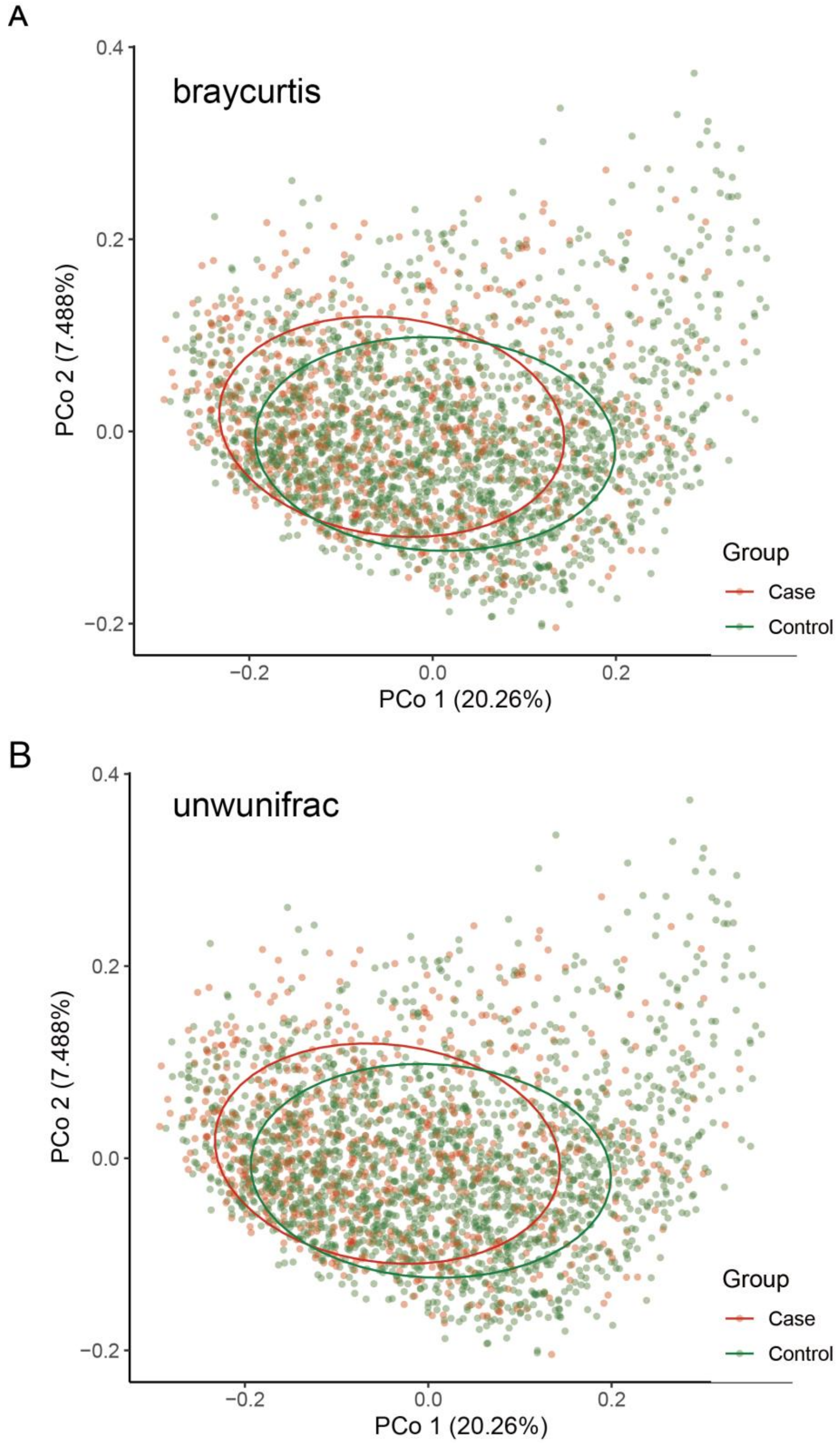

Supplementary Figure 4: Differences in bacterial taxon Distribution Between Control and Control groups.

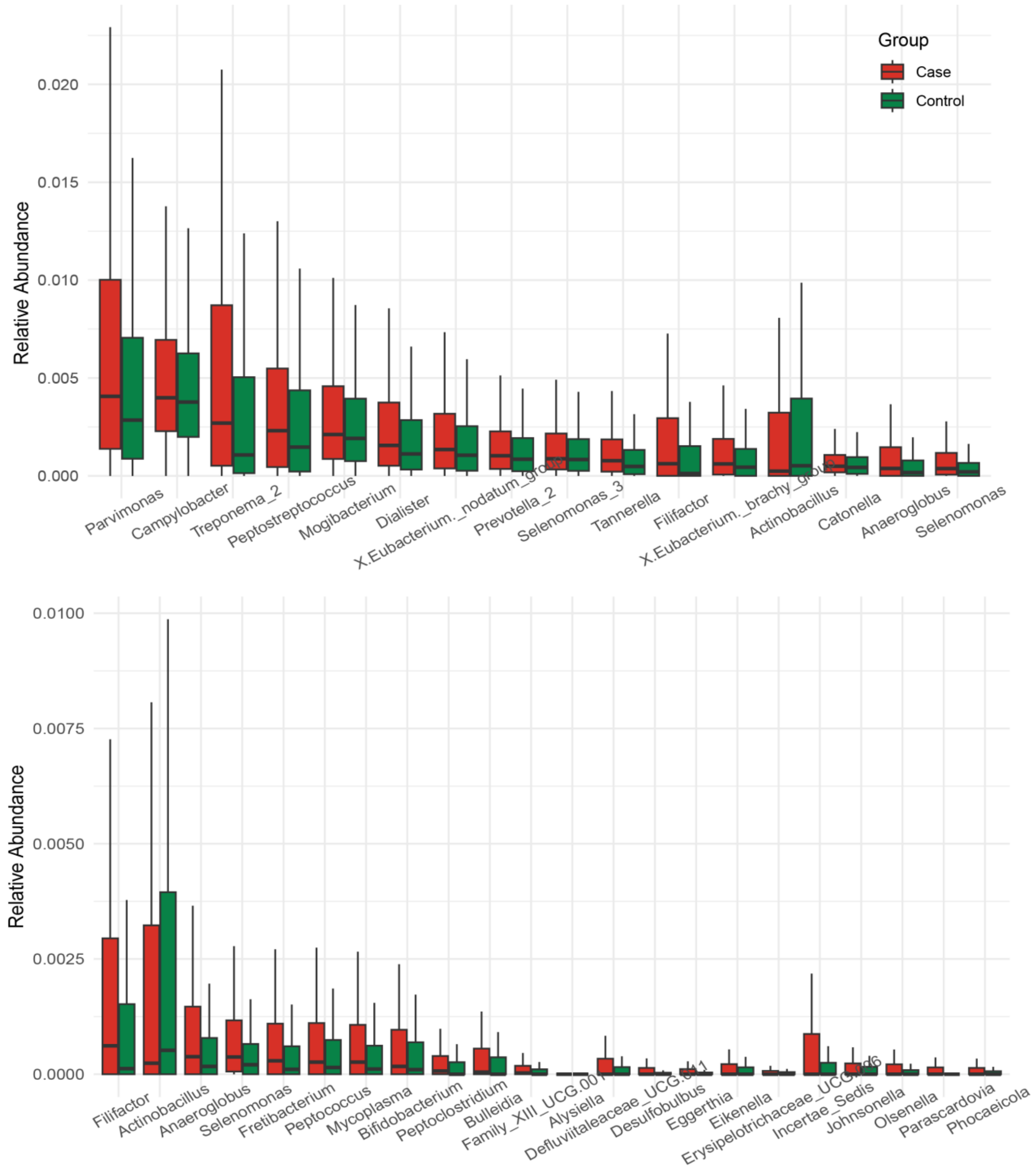

Supplementary Figure 5: Species composition comparisons between Case and Control groups.

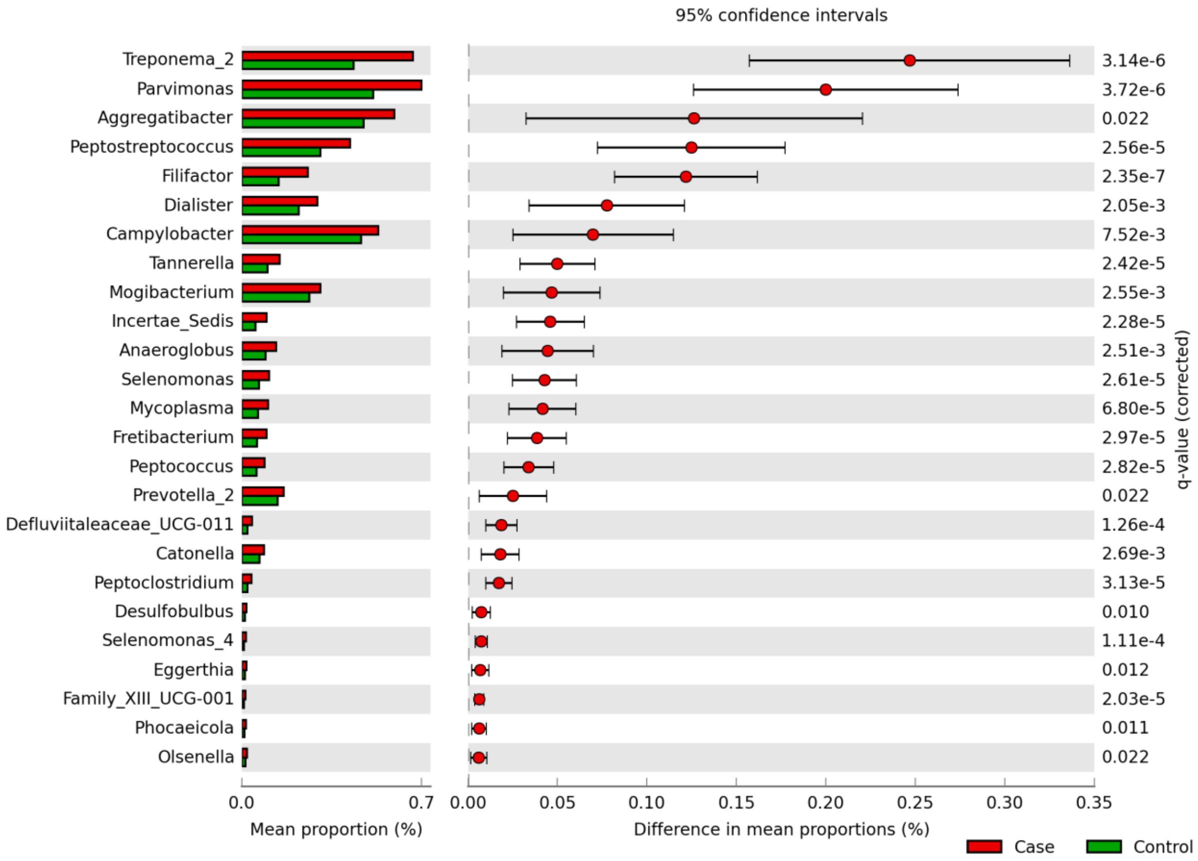

Supplement: Supplementary file 2 [file AOS-85-45662-s2.pdf]
